# Egr2-guided histone H2B monoubiquitination is required for peripheral nervous system myelination

**DOI:** 10.1093/nar/gkaa606

**Published:** 2020-07-16

**Authors:** Hannah M Wüst, Amélie Wegener, Franziska Fröb, Anna C Hartwig, Florian Wegwitz, Vijayalakshmi Kari, Margit Schimmel, Ernst R Tamm, Steven A Johnsen, Michael Wegner, Elisabeth Sock

**Affiliations:** Institut für Biochemie, Emil-Fischer-Zentrum, Friedrich-Alexander-Universität Erlangen-Nürnberg, Fahrstrasse 17, D-91054 Erlangen, Germany; Institut für Biochemie, Emil-Fischer-Zentrum, Friedrich-Alexander-Universität Erlangen-Nürnberg, Fahrstrasse 17, D-91054 Erlangen, Germany; Institut für Biochemie, Emil-Fischer-Zentrum, Friedrich-Alexander-Universität Erlangen-Nürnberg, Fahrstrasse 17, D-91054 Erlangen, Germany; Institut für Biochemie, Emil-Fischer-Zentrum, Friedrich-Alexander-Universität Erlangen-Nürnberg, Fahrstrasse 17, D-91054 Erlangen, Germany; Department of General, Visceral, and Pediatric Surgery, University Medical Center Göttingen, D-37075 Göttingen, Germany; Department of General, Visceral, and Pediatric Surgery, University Medical Center Göttingen, D-37075 Göttingen, Germany; Institut für Humananatomie und Embryologie, Universität Regensburg, Universitätsstrasse 31, D-93053 Regensburg, Germany; Institut für Humananatomie und Embryologie, Universität Regensburg, Universitätsstrasse 31, D-93053 Regensburg, Germany; Department of General, Visceral, and Pediatric Surgery, University Medical Center Göttingen, D-37075 Göttingen, Germany; Gene Regulatory Mechanisms and Molecular Epigenetics Lab, Division of Gastroenterology and Hepatology, Mayo Clinic, 200 First St SW, Rochester, MN, USA; Institut für Biochemie, Emil-Fischer-Zentrum, Friedrich-Alexander-Universität Erlangen-Nürnberg, Fahrstrasse 17, D-91054 Erlangen, Germany; Institut für Biochemie, Emil-Fischer-Zentrum, Friedrich-Alexander-Universität Erlangen-Nürnberg, Fahrstrasse 17, D-91054 Erlangen, Germany

## Abstract

Schwann cells are the nerve ensheathing cells of the peripheral nervous system. Absence, loss and malfunction of Schwann cells or their myelin sheaths lead to peripheral neuropathies such as Charcot-Marie-Tooth disease in humans. During Schwann cell development and myelination chromatin is dramatically modified. However, impact and functional relevance of these modifications are poorly understood. Here, we analyzed histone H2B monoubiquitination as one such chromatin modification by conditionally deleting the Rnf40 subunit of the responsible E3 ligase in mice. Rnf40-deficient Schwann cells were arrested immediately before myelination or generated abnormally thin, unstable myelin, resulting in a peripheral neuropathy characterized by hypomyelination and progressive axonal degeneration. By combining sequencing techniques with functional studies we show that H2B monoubiquitination does not influence global gene expression patterns, but instead ensures selective high expression of myelin and lipid biosynthesis genes and proper repression of immaturity genes. This requires the specific recruitment of the Rnf40-containing E3 ligase by Egr2, the central transcriptional regulator of peripheral myelination, to its target genes. Our study identifies histone ubiquitination as essential for Schwann cell myelination and unravels new disease-relevant links between chromatin modifications and transcription factors in the underlying regulatory network.

## INTRODUCTION

Histone modifications are numerous and occur abundantly in chromatin. These epigenetic marks influence many aspects of DNA metabolism including transcription, replication and repair. Monoubiquitination of histone H2B at lysine 120 (H2Bub1) is one of them (1). The presence of H2Bub1 immediately downstream of the transcriptional start of genes is predominantly associated with active gene transcription. It promotes di- and trimethylation of lysine 4 and lysine 79 of histone H3 and increases RNA polymerase II processivity by recruiting transcription elongation complexes such as PAF1 and pTEFb (1–3). However, recent data suggest that H2Bub1 function is dependent on the epigenetic context and that its presence on enhancers may also repress transcription (4,5). In addition to its effects on transcription in healthy cells, H2Bub1 has been implicated in DNA damage response downstream of protein kinase ATM and DNA repair (6–8).

H2Bub1 is a reversible histone mark that is predominantly introduced by the heterotetrameric Rnf40/Rnf20 E3 ligase complex (1). The Rnf40/Rnf20 complex has been shown to interact with a number of transcription factors including p53, androgen receptor, estrogen receptor and Isl1, and such interactions likely provide a major mechanism for recruitment to regulatory regions and region-specific H2B monoubiquitination (9–12). In line with the frequent association of H2Bub1 with an activated state of transcription, the Rnf40/Rnf20 complex has been reported to act as a transcriptional coactivator through direct interactions with transcription factors (13).

So far, H2B monoubiquitination has been mostly studied in cell culture where it is required during somatic cell reprogramming or in various stem and precursor cell populations for fate determination and consecutive differentiation (14–17). An association with a non-proliferative and differentiated state is reflected by H2Bub1 underrepresentation in tumors such as breast and prostate cancers, and a putative tumor suppressor function of the Rnf40/Rnf20 complex (18).

In contrast, very little information exists on the role of H2Bub1 and the Rnf40/Rnf20 complex during development of multicellular organisms. Here, we used Cre-dependent conditional gene deletion in the mouse to remove *Rnf40* selectively from Schwann cells (SCs), the main glial cell type in the peripheral nervous system (PNS) of vertebrates (19). A central task of these SCs is the formation of myelin sheaths around axons as a means of nutritional support and a prerequisite for saltatory conduction and fast impulse propagation along the nerve. In the absence of Rnf40, H2B monoubiquitination was strongly reduced in SCs. Apart from mild axonal sorting defects, embryonal SC development was largely undisturbed. However, postnatal induction of terminal differentiation, myelin production and myelin maintenance were all strongly affected leading to a severe peripheral neuropathy in these mice. We furthermore show that an altered balance between essential transcription factors in the SC regulatory network and misregulation of SC differentiation genes are crucial contributors to the observed hypomyelination. Mechanistically, Rnf40 directly interacts with Egr2 (also known as Krox20), the key transcriptional regulator of peripheral myelin formation and maintenance (20). Failure of Egr2 to recruit the Rnf40/Rnf20 E3 ligase to its target genes reduces expression of genes associated with myelin and lipid biosynthesis and increases expression of transcriptional antagonists of Egr2, thereby leading to the observed phenotype.

## MATERIALS AND METHODS

### Transgenic mice

Standard mouse housing conditions (continuous access to food and water, 12:12 h light–dark cycles etc.) and experiments were in accordance with animal welfare laws and relevant ethical regulations (approval by Veterinäramt Stadt Erlangen and Regierung von Unterfranken as responsible local committee and government body). Floxed alleles for *Rnf40* (4,21) were combined in mice with a *Dhh::Cre* transgene (22) to delete Rnf40 in developing SCs of *Rnf40^ΔSC^* mice. In some *Rnf40^ΔSC^* mice, a floxed *Sox2* allele (23,24) was additionally crossed in for simultaneous deletion. Genotyping was as described (21–24). Embryos were obtained at E12.5, E15.5, E16.5 and E18.5, sciatic nerves at P0, P5, P14, P28, P56 and P168 from male and female mice with relevant genotypes. Transgenic and control mice were on a mixed C3H × C57Bl/6J background.

### Tissue stainings

Immunohistochemical stainings were performed on transverse sections of the trunk region of mouse embryos, postnatal sciatic nerves or on teased nerve fibers (25). Preparation of tissues involved fixation in 4% paraformaldehyde, transfer to 30% sucrose, freezing in Tissue Freezing Medium (Leica) and cryotome sectioning at 10μm thickness (26). For stainings, the following primary antibodies were used: chicken anti-Mpz antibodies (Aves Labs, #PZO, Lot #PZO8767965, 1:500 dilution), goat anti-Sox2 antiserum (Santa Cruz, #sc17319, Lot #I1115, 1:500 dilution), goat anti-Sox10 antiserum (home-made, generated against a bacterially expressed and purified peptide corresponding to amino acids 181–233 and 308–400 of rat Sox10 according to accession number NM_019193.2, validated on control and knockout tissue, 1:3000), guinea pig anti-Sox10 antiserum (home-made, validated on control and knockout mouse tissue, 1:1000 dilution) (27), guinea pig anti-Egr2 antiserum (home-made, validated on mouse sciatic nerve, 1:1000 dilution) (25), rabbit anti-Egr2 antiserum (Covance, #PRB-236P, Lot #D13BF00486, 1:200 dilution), rabbit anti-Rnf40 monoclonal (Abcam, #ab191309, Lot #11974, 1:100 dilution), rabbit anti-Rnf20 monoclonal (Cell signaling, #11974, Lot #1, 1:100 dilution), rabbit anti-Oct6 antiserum (home-made, validated on control and knockout mouse tissue, 1:2000 dilution) (25), rabbit anti-Nav1.6 antiserum (Alomone Labs, #ASC-009, Lot#ASC009AN2425, 1:50 dilution), rabbit anti-Caspr antiserum (Abcam, #ab34151, Lot #GR86230, 1:1000 dilution), rabbit anti-Iba1 antiserum (Wako, #019–19741, Lot #SAE6921, 1:250 dilution), rabbit anti-Ki67 antiserum (Thermo Fisher Scientific, #RM-9106, Lot#9106S906D, 1:500 dilution), rat anti-Ki67 antiserum (Thermo Fisher Scientific, #14–5698-82, Lot #2056928, 1:500 dilution), rat anti-MBP monoclonal (Bio-Rad, #MCA409S, Lot #210610, 1:300 dilution), mouse anti-H2Bub1 monoclonal (gift of D. Eick, Helmholtz Zentrum München, 1:5 dilution) (9). Secondary antibodies were coupled to Cy3 (Dianova, 1:200 dilution), Cy5 (Dianova, 1:200 dilution) or Alexa488 (Molecular Probes, 1:500 dilution) fluorescent dyes. Nuclei were counterstained with DAPI. TUNEL was performed according to the manufacturer's protocol (Chemicon). Paraphenylene-2,6-diamine (PPD) staining of sciatic nerve sections was as described (28). Stainings were documented with a Leica DMI 6000B inverted microscope equipped with a DFC 360FX camera or with a Zeiss inverted Axio Observer 7 with ApoTome.2 equipped with an Axiocam 503 and a Colibri 7 LED light source.

### Electron microscopy

Sciatic nerves of control and genetically altered mice were dissected at P14, P28 and P56, followed by fixation in cacodylate-buffered fixative containing 2.5% paraformaldehyde and 2.5% glutaraldehyde, incubation in cacodylate-buffered 1% osmium ferrocyanide, dehydration, embedding in Epon resin, transverse sectioning and staining with uranyl acetate and lead citrate. 50 nm sections were examined with a Zeiss Libra electron microscope. From electron microscopic pictures, the number of unmyelinated axons bigger than 1μm and the g ratio of myelinated axons were determined.

### RNA-Seq, ChIP-Seq and bioinformatic analysis

Total RNA was prepared from pools of three sciatic nerves obtained at P14 from control and Rnf40^ΔSC^ mice in triplicates. The quality of samples was evaluated by RNA agarose gel electrophoresis. 500 ng total RNA was used for library preparation with the NEXTFLEX^®^ Rapid Directional RNA-Seq Kit (Bioo Scientific) according to the manufacturer's instructions. After pooling, libraries were sequenced on an Illumina HiSeq 2500 platform at the Next Generation Sequencing Integrative Genomics Core Unit (University Medical Center Göttingen, Germany) and aligned to mouse genome mm9 using TOPHAT (v2.1.1). Unique mapped reads were assigned to genomic features using HTSeq-counts (v0.9.1). Differential expression analysis was carried out using the DESeq2 package (v1.10.1) with default parameters in the R statistical environment. The mRNA sequencing files are accessible on the Gene Expression Omnibus (GEO) platform under accession number GSE146629.

GO analysis of genes up- and downregulated in SCs of the Rnf40^ΔSC^ sciatic nerve at P14 was performed using the Gene Ontology enrichment, analysis and visualization tool GOrilla (http://cbl-gorilla.cs.technion.ac.il/) or the Database for Annotation, Visualization and Integrated Discovery tool DAVID (https://david.ncifcrf.gov/) with comparable results. In most cases, results from GOrilla are shown. Redundant GO terms were removed by subsequent analysis with ReviGO (http://revigo.irb.hr/). Gene signatures consequent to RNF40 loss were identified using the Gene Set Enrichment Analysis (GSEA) tool from the Broad Institute (http://software.broadinstitute.org/gsea/index.jsp).

ChIP-Seq experiments were performed in biological triplicates with anti-H2Bub1 (Cell signaling, #5546, 1.5μg/IP), anti-H3K27ac (Diagenode, # C15410196, 1μg/IP) and anti-H3K27me3 (Diagenode, # C15410195, 1μg/IP) antibodies. For each sample, four sciatic nerves were used from control or Rnf40^ΔSC^ mice at P14. ChIP was performed as described (29). Briefly, sciatic nerve chromatin was cross-linked in 1% formaldehyde at room temperature and sheared to fragments of ∼100–250 bp in a Bioruptor (Diagenode). Sheared, quantified and pre-cleared chromatin was incubated with specific antibodies or control IgG (Abcam, #Ab37415) and precipitated using BSA-pretreated protein A sepharose beads (GE Healthcare, GE17-0780-01). Precipitated chromatin and input were de-crosslinked and DNA was purified by proteinase K treatment, phenol/chloroform extraction and ethanol precipitation. The resulting DNA was used for library generation using the MicroPlex Library Preparation Kit (Diagenode) according to the manufacturer's instructions. Size and quality controls were performed with a Bioanalyzer 2100 (High Sensitivity DNA assay). Afterwards, single-end sequencing (51 bp) was performed on a HiSeq 2000 Illumina platform by the Transcriptome and Genome Analysis Laboratory (University Medical Center Göttingen, Germany). Approximately 20 million reads were generated per sample. Data are deposited in GEO under accession number GSE146645.

Reads were mapped to the mouse reference genome (GRCm38/mm9) using Bowtie2 (version 2.3.4.2). After removing PCR duplicates with RmDup (version 2.0.1) mapped reads were converted to BAM format via SAMtools (version 1.1.2). BAM files were further processed using the BamCoverage tool within the deeptools software (version 3.0.2.0) and normalized to 1×. For peak calling Model-based Analysis of ChIP-Seq (MACS2) (version 2.1.1.20160309.0) was used with input samples set as background and default parameters (*q*-value < 0.05). Single gene profiles were visualized in the Integrated Genome Viewer (IGV; version 2.3.98). To illustrate the average distribution of histone modifications 5 kb up- and downstream of the TSS of genes with H2B monoubiquitination, heatmaps and plot profiles were generated using computeMatrix (Galaxy Version 3.3.2.0.0) followed by plotHeatmap (Galaxy Version 3.3.2.0.1). To detect ChIP-Seq peaks with differences in signal intensity in control and *Rnf40^ΔSC^* chromatin, differential binding analysis was performed for all peak regions called with MACS2 in control and *Rnf40^ΔSC^* chromatin using DiffBind (version 2.6.6).

To assign regions significantly depleted for H2Bub1 in *Rnf40^ΔSC^* chromatin (*P*-value < 0.05) to specific genes, genomic positions of depleted regions were compared and matched to the gene coordinates downloaded from the UCSC Table Browser (UCSC genes GRCm38/mm9) using intersect (version 1.0.0). Subsequently, the identified H2Bub1-depeleted genes were overlapped with the up- or downregulated genes in *Rnf40^ΔSC^* sciatic nerves using the Venn diagram tool of the PBE homepage (http://bioinformatics.psb.ugent.be/webtools/Venn/). The retrieved sets of genes with combined H2Bub1 depletion and up- or downregulated expression were submitted to a combination of GO analysis and ReviGO as described.

To detect potential effects of H2B monoubiquitination on the expression of Egr2 target genes, a list of direct Egr2 target genes was compiled by overlapping the published list of differentially expressed genes in Egr2 Lo/Lo mice (30) (after determination of their chromosomal position in rn4 and expanding each locus by 50 kb on either side) to the published Egr2 ChIP-Seq peaks from sciatic nerves of P14 old rats (31) using bedtools Intersect intervals (v.2.29.0) by default overlap of 1bp. The obtained list of direct Egr2 target genes was compared to genes depleted for H2Bub1 in Rnf40*^ΔSC^* chromatin using the Venn diagram tool. H2Bub1-depleted direct Egr2 target genes and Egr2 target genes with unchanged H2Bub1 status underwent GO analysis and subsequent ReviGO.

### Cell culture, plasmids and transfections

For primary SC culture, sciatic nerves and brachial plexi of early postnatal rat pups were dissected, incubated in trypsin/collagenase solution for 1 h and dissociated by up-and-down pipetting. Dissociated cells were grown under proliferative conditions in DMEM/F12 supplemented with 0.0035% bovine serum albumin (Life technologies, #15260), 16 μg/ml putrescine (Sigma, #P5780), 100 μg/ml transferrin (Sigma, #T4515), 38 ng/ml dexamethasone (Sigma, #D8893), 10 ng/ml T3 (Sigma, #T6397), 400 ng/ml T4 (Sigma, #T1775), 60 ng/ml progesterone (Sigma, #P8783), 160 ng/ml selenium (Sigma, #S5261) 20 ng/ml neuregulin 1 (Peprotech, #100-03) and 50 μM di-butyryl-cAMP (Sigma, #D0627). For differentiation, di-butyryl-cAMP concentration was increased to 1 mM and 5.7 μg/ml insulin (Sigma, #I5500) was added.

HEK293T cells (obtained from ATCC, authenticated by PCR) were grown in DMEM supplemented with 10% fetal calf serum and transfected with pCMV5-based expression plasmids for Rnf40 (accession number NM_014771), Rnf20 (accession number NM_019592), Egr2 (accession number NM_053633) and various variants at 10 μg per 10 cm plate using polyethylenimine. Further information on plasmids is provided in the Supplementary Tables.

Mouse Neuro2a neuroblastoma cells (obtained from ATCC, authenticated by PCR) were transfected with various combinations of pCMV5-Egr2 and pSuper-based expression plasmids for a Rnf40-specific (5′-GATCCCCGGCCAGATTACACTCACATGTTCAAGAGACATGC TGAGTGTAATCTGGCCTTTTTC-3′) and a scrambled shRNA using Superfect reagent (Qiagen) (32). An amount of 7.5 μg was used for each reporter plasmid per 10 cm plate. Overall amounts of plasmid were kept constant by adding empty pCMV5 or pSuper where necessary. After 72 h in culture, transfected cells were isolated by FACS using green fluorescent protein produced from pSuper plasmids.

### Quantitative RT-PCR (qRT-PCR) analysis

For qRT-PCR, total RNA was prepared from transfected and FACS sorted Neuro2a cells using RNeasy® Micro Kit (Qiagen, #74004). After reverse transcription, samples were subjected to PCR on a CFX96 Real Time PCR System (Bio-Rad) using the primers listed in the Supplementary Tables. The melting curve of each sample was checked to ensure specificity of the amplified products. All samples were processed as technical triplicates. Data were analyzed by the ΔΔCt method.

### Extract preparation, immunoprecipitation and western blots

Whole cell extracts were prepared from differentiated primary rat SCs and transfected HEK293T cells as reported (26). For immunoprecipitation experiments, whole cell extracts from HEK293T cells and SCs were diluted in TEN buffer before addition of pre-immune sera, rabbit IgG, or one of the following antibodies: rabbit anti-Egr2 antiserum (home-made, generated against a bacterially expressed and purified peptide corresponding to amino acids 28–166 of mouse Egr2 according to accession number NM_010118.3, validated on mouse sciatic nerve tissue), guinea pig anti-Egr2 antiserum, rabbit anti-Rnf40 monoclonal or rabbit anti-Rnf20 monoclonal. Incubation was overnight at 4°C in the presence of protein A sepharose beads (GE Healthcare, #17-0780-01, Lot #10254134) before samples were processed and precipitated proteins were eluted from beads as described (33,34).

After size-separation of extracts and immunoprecipitates on 8–12% polyacrylamide-SDS gels and western blotting onto nitrocellulose membranes, the following antibodies and detection reagents were used: guinea pig anti-Egr2 antiserum (1:20 000 dilution), rabbit anti-Egr2 antiserum (1:3000 dilution), mouse anti-myc monoclonal (Cell signaling, #2276, Lot #0024, 1:10 000 dilution), mouse anti-HA monoclonal (Santa Cruz, #sc-7392, Lot # I0717, 1:5000 dilution), rabbit anti-Rnf40 monoclonal (1:2000 dilution), rabbit anti-Rnf20 monoclonal (1:4000 dilution), horseradish peroxidase coupled to protein A (Zymed, #10-1023, Lot #20873065, 1:3000 dilution) and horseradish peroxidase-labelled anti-mouse IgG antibody (KPL, #074-1506, Lot #130535, 1:3000 dilution). Detection was by chemiluminescence using ECL reagent. Uncropped western blot images are depicted in Supplementary Figures S9 and S10.

### Quantifications and statistical analysis

Results from independent animals, experiments or separately generated samples were treated as biological replicates. Sample size was *n* ≥ 3 for all molecular biology experiments and experiments with mice as common for this kind of study. No data were excluded from the analysis. Randomization was not possible. Investigators were not blinded in animal experiments. Fluorescence intensities were quantified using Fiji ImageJ software, W.S. Rasband, U.S. National Institutes of Health, Maryland, USA. GraphPad Prism6 software, La Jolla, CA, USA was used to determine whether differences in cell and axon numbers, *g*-ratios or transcript levels were statistically significant by ANOVA or unpaired, two-tailed Student's *t* tests (*P ≤ 0.05; **P ≤ 0.01, ***P ≤ 0.001). The data met the assumptions of the chosen test. Variance between statistically compared groups was similar.

## RESULTS

### Rnf40 expression in SCs

**Figure 1.**
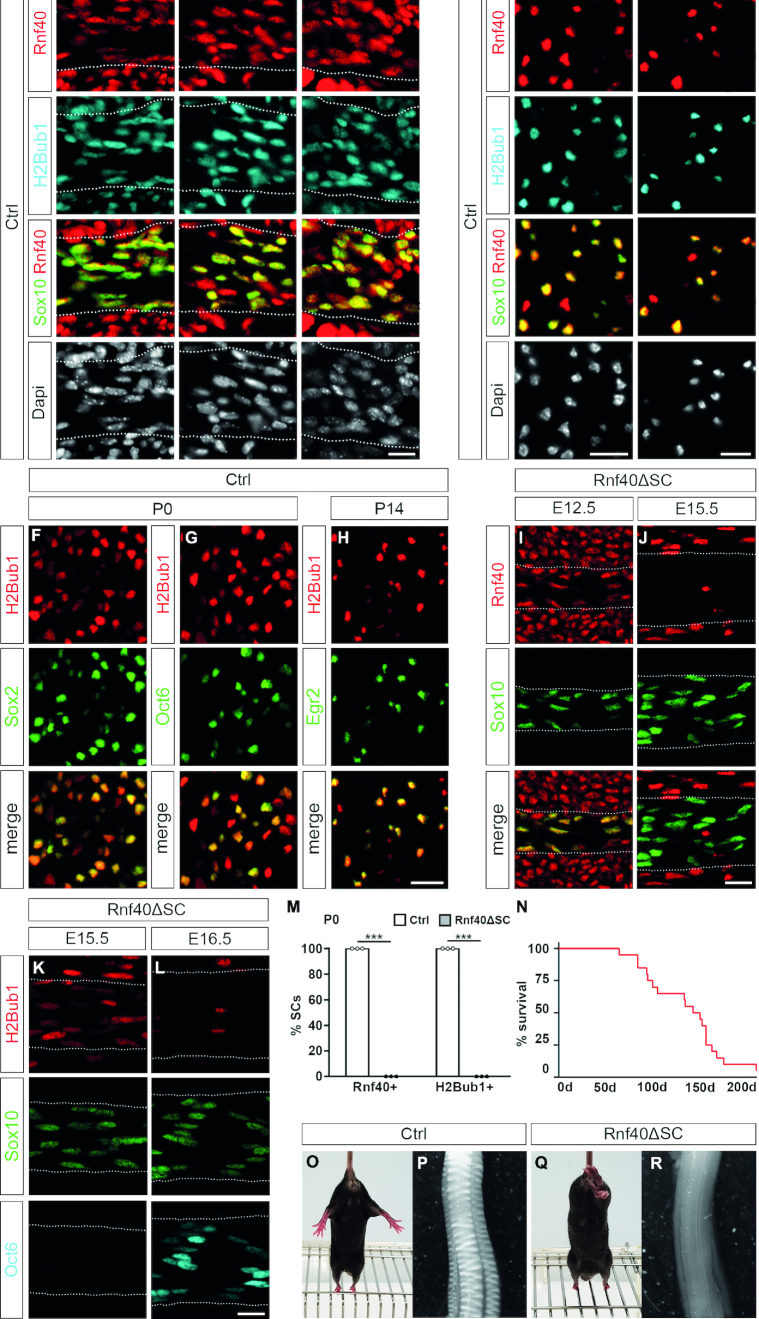
Peripheral neuropathy resulting from Rnf40 deletion and H2Bub1 loss in SCs. (**A**–**E**) Temporal occurrence of Rnf40 and H2Bub1 in SCs of spinal (A–C) and sciatic (D, E) nerves from control mice at E12.5 (A), E15.5 (B), E18.5 (C), P5 (D) and P56 (E) as determined by co-immunofluorescence studies with antibodies against Rnf40 (red), H2Bub1 (cyan) and Sox10 (green). Nerves are demarcated by dotted lines. (**F**–**H**) Stage-specific occurrence of H2Bub1 in SCs as determined by co-immunofluorescence studies with antibodies against Sox2 (F, P0), Oct6 (G, P0) and Egr2 (H, P14). (**I**–**L**) Deletion kinetics of Rnf40 (I, J) and H2Bub1 (K, L) in SCs from spinal nerves of *Rnf40^ΔSC^* mice as determined by immunofluorescence studies with antibodies against Rnf40 (red in I, J), H2bub1 (red in K, L), Sox10 (green) and Oct6 (cyan) at E12.5 (I), E15.5 (J, K) and E16.5 (L). (**M**) Quantification of the relative numbers of Rnf40- and H2Bub1-positive SCs in sciatic nerves of control (Ctrl, white bars) and *Rnf40^ΔSC^* (gray bars) mice at P0 following immunohistochemistry. SCs were identified by Sox10 staining (*n* = 3; mean values ± SEM). (**N**) Survival curve of *Rnf40^ΔSC^* mice (red line, *n* = 20) during the first 6 months of life. All controls stayed alive during the analyzed period. (**O**–**R**) Hindlimb clasping phenotype (O, Q) and sciatic nerve hypomyelination (P, R) in control (O, P) as compared to *Rnf40^ΔSC^* (Q, R) mice at P28. Scale bars: 15 μm. Statistical significance was determined by unpaired, two-tailed Student's *t*-test (***P ≤ 0.001). Exact values are provided in the Supplementary Tables.

Considering the widespread occurrence of H2Bub1 in many cell types, it seemed likely that H2Bub1 and the E3 ligase responsible for introducing this histone modification are present in SCs as well. To characterize the SC expression pattern, we performed immunohistochemical stainings with antibodies directed against H2Bub1, Rnf40 and Sox10, a transcription factor that serves as a SC lineage marker (35). During embryonic development we focused on spinal nerve and postnatally on sciatic nerve. Both H2Bub1 and Rnf40 were present in Sox10-positive SCs of spinal nerves at E12.5, E15.5 and E18.5 (Figure 1A–C). H2Bub1 and Rnf40 also co-localized in Sox10-positive SCs of the postnatal sciatic nerve at all time points of analysis from the day of birth until 8 weeks of age (Figure 1D, E and Supplementary Figure S1A–C). Co-immunohistochemical stainings with stage-specific SC markers furthermore confirmed H2B monoubiquitination in Sox2-positive immature SCs (36), Oct6-positive promyelinating SCs (37) and Egr2-positive myelinating SCs (20), and thus at all stages of SC development (Figure 1F–H). H2Bub1 and Rnf40 also occurred in other cell types of the peripheral nerve. An identical expression pattern was also detected for Rnf20 (Supplementary Figure S1D–K).

### Peripheral hypomyelination in mice with SC-specific Rnf40 deletion

Both Rnf40 and Rnf20 have to be present for E3 ligase activity (1). Therefore, Rnf40 inactivation should be sufficient to prevent H2Bub1 formation. To study the role of H2Bub1 in SCs and their development, we combined a floxed *Rnf40* allele, in which exons 3 and 4 are flanked by loxP sites (4,21), with a *Dhh::Cre* transgene (22). The *Dhh::Cre* transgene was chosen because of its early and restricted expression in the Schwann cell lineage. Use of an even earlier, broadly expressed *Sox10::Cre* (38) had shown before that Rnf40 is not required for Schwann cells to develop into the *Dhh::Cre* expressing precursor state (data not shown). In these *Rnf40^ΔSC^* mice, Cre is selectively induced during embryogenesis at late SC precursor to early immature SC stage and continues to be expressed in the SC lineage. As a consequence, Rnf40 was still detected in all cells of spinal nerves at E12.5, but absent from Sox10-positive SCs at E15.5 (Figure 1I, J). Other cells in peripheral nerve and surrounding tissue remained Rnf40-positive. At E15.5, H2Bub1 had also strongly decreased in spinal nerve SCs of *Rnf40^ΔSC^* mice (Figure 1K). However, residual amounts were still present but had disappeared from SC chromatin by E16.5 (Figure 1L). Its loss preceded induction of Oct6, which appeared around E16.5 in spinal nerves of control and *Rnf40^ΔSC^* mice, and is a marker and transcriptional regulator of the promyelinating stage (Figure 1L). We conclude from these findings that Rnf40 is lost from SCs in *Rnf40^ΔSC^* mice in the early immature stage, whereas H2Bub1 persists at residual amounts into the late immature stage. The H2Bub1 modification thus has a slower turnover than the Rnf40/Rnf20 complex. At birth, SC-specific deletion of Rnf40 and H2Bub1 was complete in sciatic nerves (Figure 1M). Our results confirm the Rnf40/Rnf20 complex as the major H2B ubiquitination activity in SCs.

*Rnf40^ΔSC^* mice were born at normal Mendelian ratios and indistinguishable from their littermates until postnatal day (P) 14. However, with increasing age *Rnf40^ΔSC^* mice developed progressive gait abnormalities, ataxia and tremor. Hindlimb clasping was observed (Figure 1O and Q). By P56, *Rnf40^ΔSC^* mice had severe problems to move around in the cage and did not reach normal age (Figure 1N). By macroscopic inspection, the dissected sciatic nerve of *Rnf40^ΔSC^* mice was translucent at P28, whereas it appeared opaque in control mice and exhibited a typical banded pattern (Figure 1P and R). These findings point to a severe hypomyelinating phenotype and a SC defect in *Rnf40^ΔSC^* mice.

### SC characteristics in *Rnf40^ΔSC^* nerves

Sciatic nerve SCs undergo differentiation during the first two postnatal weeks. To identify potential consequences of Rnf40 inactivation on SC development, myelin formation and myelin maintenance, we quantified and characterized SCs by immunohistochemical stainings in *Rnf40^ΔSC^* sciatic nerves during the first two months starting at the day of birth by focusing on the tibial branch at upper thigh levels.

**Figure 2.**
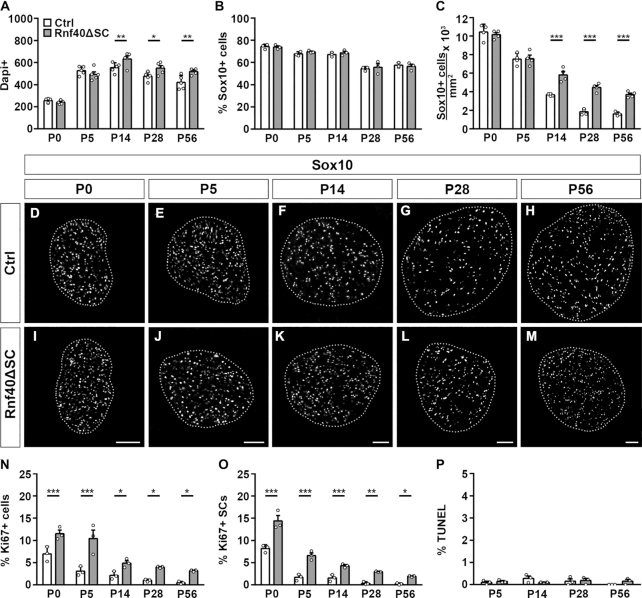
SC numbers and characteristics in sciatic nerves of *Rnf40^ΔSC^* mice. (**A**–**C**) Comparison of the number of total cells (A) as well as the relative contribution of SCs to the overall cell population (B) and SC density (C, as determined by the number of SCs per mm^2^) in sciatic nerve sections of control (Ctrl, white bars) and *Rnf40^ΔSC^* (gray bars) mice at P0, P5, P14, P28 and P56 by quantification of nerve sections stained with DAPI and antibodies against Sox10 (*n* = 3–5; mean values ± SEM). (**D**–**M**) Immunohistochemical stainings of sciatic nerve sections of control (D–H) and *Rnf40^ΔSC^* (I–M) mice at P0 (D, I), P5 (E, J), P14 (F, K), P28 (G, L) and P56 (H, M) with antibodies directed against Sox10. Sections were placed on a black background and are surrounded by a dotted line. Scale bars: 50μm. (**N**–**P**) Comparison of the number of Ki67-positive proliferating cells (N), the percentage of Ki67-positive proliferating SCs (O) and total TUNEL-labled cells (P) in sciatic nerve sections of control (Ctrl, white bars) and *Rnf40^ΔSC^* (gray bars) mice at P0, P5, P14, P28 and P56 (*n* = 3; mean values ± SEM). Statistical significance was determined by unpaired two-tailed Student's *t*-tests (*P ≤ 0.05; **P ≤ 0.01; ***P ≤ 0.001). Exact values are provided in the Supplementary Tables.

During the period of analysis, total cell numbers in control sciatic nerves increased from approximately 256 ± 9 cells at P0 to 420 ± 27 cells at P56 per nerve section as determined by DAPI staining (Figure 2A; Supplementary Figure S2A, B). During the first week, cell numbers were similar in sciatic nerves of *Rnf40^ΔSC^* nerves. However, by the end of the second week, cell numbers in sections from sciatic nerves of *Rnf40^ΔSC^* mice exhibited a small, but statistically significant increase of ∼14% compared to control. Cell numbers increased further over the period of analysis and were 24% higher at P56.

In control sciatic nerves, Sox10-positive SCs constituted ∼70–75% of all cells at P0 and were present at a density of 10.4 × 10^3^ cells per mm^2^ (Figure 2B and C). At P56, SCs represented ∼55–60% of all cells in the sciatic nerve and their density had decreased to 1.6 × 10^3^ cells/mm^2^ as a consequence of the myelination process (Figure 2B and C). The contribution of Sox10-positive SCs to the overall cell population in the sciatic nerve was comparable at all times in control and *Rnf40^ΔSC^* mice (Figure 2B). However, SC density was 1.6- to 2.3-fold higher in sciatic nerves of *Rnf40^ΔSC^* mice from P14 onward (Figure 2C–M). Considering the increased cell number in sciatic nerve of *Rnf40^ΔSC^* mice and the similar contribution of SCs to the total population, SCs are partly responsible for the higher total cell number. Supporting this conclusion, numbers of Ki67-positive cells and the percentage of Ki67-positive SCs were both increased at all analyzed time points in *Rnf40^ΔSC^* sciatic nerves (Figure 2N and O; Supplementary Figure S2C, D). Rates of Ki67-positive SCs decreased steadily in both genotypes. However, the number of Ki67-positive SCs decreased from 8.2 ± 0.5% at P0 to 0.2 ± 0.1% at P56 in sciatic nerves of control mice, whereas it decreased from 14.4 ± 1.2% to 2.0 ± 0.1% in sciatic nerves of *Rnf40^ΔSC^* mice. By contrast, dying cells as measured by TUNEL were few and did not substantially differ between the two genotypes throughout the period of analysis (Figure 2P; Supplementary Figure S2E, F).

**Figure 3.**
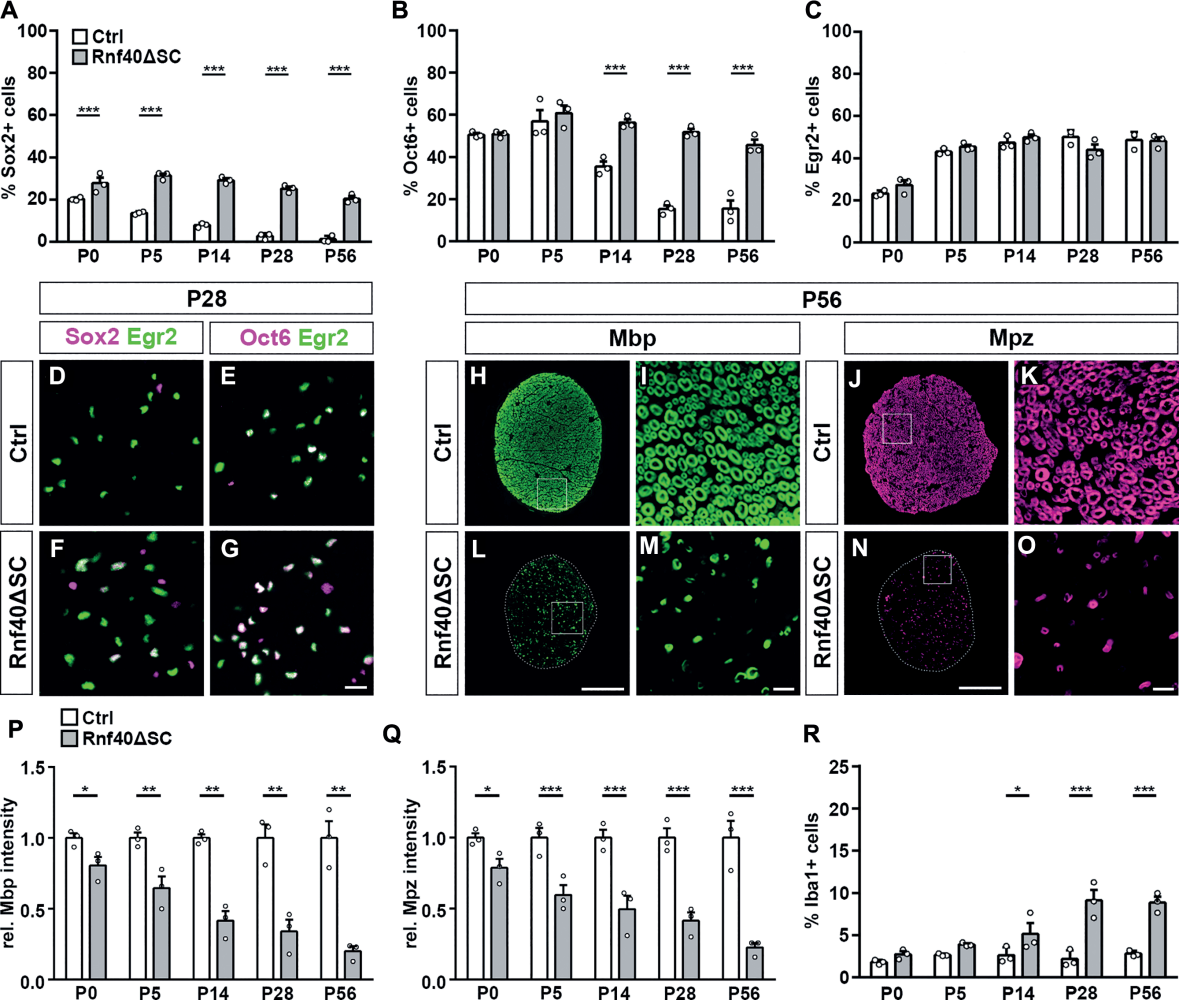
SC development in sciatic nerves of *Rnf40^ΔSC^* mice. (**A**–**C**) Comparison of the relative contribution of Sox2+ immature (A), Oct6+ promyelinating (B) and Egr2+ myelinating SCs (C) to the SC population in sciatic nerve sections of control (Ctrl, white bars) and *Rnf40^ΔSC^* (gray bars) mice at P0, P5, P14, P28 and P56 (*n* = 3; mean values ± SEM). (**D**–**O**) Immunohistochemical stainings of sciatic nerve sections of control (D, E, H–K) and *Rnf40^ΔSC^* (F, G, L–O) mice at P28 (D–G) and P56 (H–O) with antibodies directed against Egr2 (green in D–G), Sox2 (magenta in D, F), Oct6 (magenta in E, G), Mbp (green in H, I, L, M) and Mpz (magenta in J, K, N, O). Nerve sections in H,J,L,N were placed on a black background and areas marked by boxes were magnified in I, K, M, O. Scale bars:15 μm in G; 100 μm in L, N; 10 μm in M, O. (**P**, **Q**) Quantification of signal intensities for myelin proteins Mbp (P) and Mpz (Q) in sciatic nerve sections of control (Ctrl, white bars) and *Rnf40^ΔSC^* (ΔSC, gray bars) mice from P0 to P56 (*n* = 3; mean values ± SEM). (**R**) Quantification of Iba1-positive macrophages relative to the cell population in sciatic nerve sections of control (Ctrl, white bars) and *Rnf40^ΔSC^* (ΔSC, gray bars) mice from P0 to P56 (*n* = 3; mean values ± SEM). Statistical significance was determined by unpaired two-tailed Student's *t*-test (*P ≤ 0.05; **P ≤ 0.01; ***P ≤ 0.001). Exact values are provided in the Supplementary Tables.

At birth, 20.3 ± 0.5% of cells in sciatic nerves of control mice were immature SCs that expressed the stage-specific marker Sox2 (Figure 3A; Supplementary Figure S3A). At this time point, percentage and absolute number of Sox2-expressing SCs were slightly, but significantly increased in sciatic nerves of *Rnf40^ΔSC^* mice to 27.8 ± 2.6% (Figure 3A; Supplementary Figure S3B). Interestingly, the percentage of Sox2-expressing cells in sciatic nerves of *Rnf40^ΔSC^* mice remained above 20% throughout the whole period of analysis, whereas it decreased rapidly in sciatic nerves of control mice and was already <8% at P14 reflecting the rapid differentiation of SCs during the first two postnatal weeks (Figure 3A; Supplementary Figure S3A, B).

Many SCs in sciatic nerves of control mice expressed Oct6 as a marker of the promyelinating stage at birth (Figure 3B; Supplementary Figure S3C). As the promyelinating stage is a transient phase during SC differentiation, the percentage of Oct6-expressing cells in the nerve remained high during the first postnatal week and then declined. Starting from P28, the percentage of Oct6-expressing cells remained at a steady level <20%. In sciatic nerves of *Rnf40^ΔSC^* mice, the percentage of promyelinating SCs was comparable to controls throughout the first week (Figure 3B; Supplementary Figure S3D). However, instead of decreasing, Oct6-positive SCs remained high and still made up 45.6 ± 2.5% of the nerve cell population at P56.

For progression of SC differentiation, expression of the transcription factor Egr2 as the central regulator of myelination and myelin maintenance is needed. Accordingly, Egr2-positive SCs were still few at P0 in sciatic nerves of control and *Rnf40^ΔSC^* mice and represented 23–27% of all cells (Figure 3C; Supplementary Figure S3E, F). During the first postnatal week, the number of Egr2-positive myelinating SCs increased rapidly and remained stable at a high level. In contrast to other stage-specific markers, there were no significant differences in the percentage of Egr2-positive SCs between control and *Rnf40^ΔSC^* mice at any of the analyzed time points (Figure 3C; Supplementary Figure S3E, F). Egr2-expressing SCs did not contain Sox2 in control sciatic nerves at P28 (Figure 3D). Approximately one third was Oct6-positive (Figure 3E). Intriguingly, age-matched *Rnf40^ΔSC^* sciatic nerves contained a strongly increased number of SCs that expressed Egr2 in combination with Sox2 or Oct6 (Figure 3F and G).

In light of the normal Egr2, persistent Sox2, and increased Oct6 expression, we analyzed myelin gene expression and focused on Mbp and Mpz as the two main peripheral myelin proteins. In agreement with what is known from the literature, Mbp- and Mpz-positive cells were still few in control sciatic nerves at P0 but rapidly increased during the first two weeks before reaching high steady-state levels at P28 (Figure 3H–K, Supplementary Figure S4A, C). By contrast, Mbp- and Mpz-positive cells were dramatically reduced at all analyzed time points in *Rnf40^ΔSC^* nerves and remained substantially below control levels even at P56 (Figure 3L–O, Supplementary Figure S4B, D). Mbp signals were also reduced in intensity at all times in *Rnf40^ΔSC^* nerves and dropped from 80.7 ± 5.0% of control levels at P0, when there is still little myelin gene expression, to 20.0 ± 3.5% of control levels at P56 when myelination has reached a steady state in controls (Figure 3P). Mpz signals behaved identically (Figure 3Q).

Considering the dramatic reduction in myelin gene expression, we checked for signs of inflammation by counting the number of Iba1-positive macrophages. During the first week, the number of macrophages was comparable between control and *Rnf40^ΔSC^* nerves (Figure 3R; Supplementary Figure S3G, H). However, from the end of the second week onward, numbers of Iba1-positive macrophages were substantially elevated in nerves of *Rnf40^ΔSC^* mice and constituted 5.2 ± 1.3% of the nerve cell population as compared to 2.6 ± 0.6% in controls at P14 and further increased to 8.9 ± 0.7% at P56, while control levels remained constant. Iba1-positive macrophages were the second cell type that exhibited increased proliferation in *Rnf40^ΔSC^* nerves (data not shown). Rnf40-dependent alterations in SCs thus appear to trigger a secondary inflammatory response.

### Hypomyelination in *Rnf40^ΔSC^* nerves

**Figure 4.**
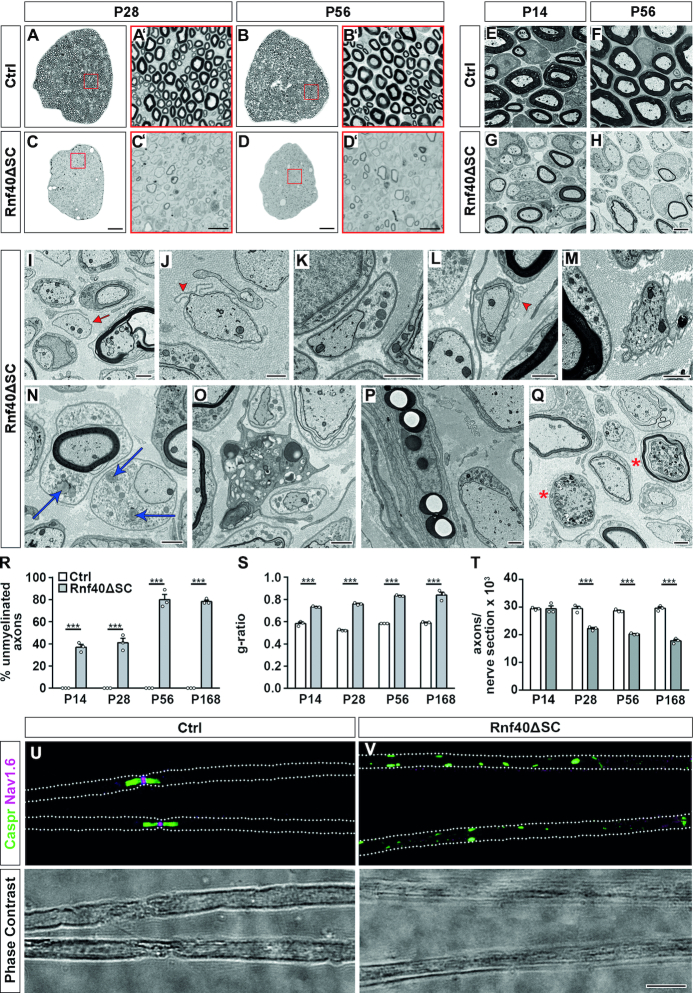
Histological and ultrastructural alterations in *Rnf40^ΔSC^* nerves. (**A**–**D**) Myelin structures visualized by PPD staining on transverse sciatic nerve sections of control (A, B) and *Rnf40^ΔSC^* (C, D) mice at P28 (A, C) and P56 (B, D). Higher magnifications are shown in A’–D’. (**E**–**Q**) Representative electron microscopic pictures of sciatic nerve sections from control (E, F) and *Rnf40^ΔSC^* (G–Q) mice at P14 (E, G), P28 (I, J, L, M, N, P) and P56 (F, H, K, O, Q) in overview (E–H) and at higher resolution (I–Q). Magnifications depict naked axons (I, red arrow), axons with residual SC-derived basal lamina (J, red arrowhead), axons partly wrapped by SCs (K), axons surrounded by a SC with partly detached basal lamina (L, red arrowhead), SCs without associated axon (M), droplet like inclusions in SCs (N, blue arrows), in activated macrophages (O), in perineurial cells (P) and degenerating axons (Q, asterisks). (**R**–**T**) Determination of the number of unmyelinated axons as percent of total axons with a diameter ≥1μm (R), mean *g*-ratio (S), number of axons with a diameter ≥1 μm per tibial branch of sciatic nerve (T) in ultrathin sciatic nerve sections of control (white bars) and *Rnf40^ΔSC^* (gray bars) mice from P14 to P168 (*n* = 3). All large caliber axons were myelinated in control mice. (**U**, **V**) Immunohistochemical staining (upper panels) and corresponding phase contrast photographs (lower panels) of teased fibers obtained from sciatic nerves of control (U) and *Rnf40^ΔSC^* (V) mice at P28 with antibodies against the paranodal marker Caspr (green) and the nodal marker Na_v_1.6 (magenta). Scale bars: 50 μm in C, D, 10 μm in C’, D’, V, 2 μm in H, 1 μm in I–Q. Statistical significance was determined by unpaired, two-tailed Student's *t*-test (*P ≤ 0.05; ***P ≤ 0.01; *P* ≤ 0.001). Exact values are provided in the Supplementary Tables.

Next, we performed a histological analysis of transversely sectioned *Rnf40^ΔSC^* nerves in comparison to control nerves. PPD stainings revealed a dramatic reduction in the number of myelinated axons in *Rnf40^ΔSC^* nerves at P28 and P56 (Figure 4A–D). Existing myelin sheaths furthermore appeared to be much thinner.

To obtain a higher resolution, we analyzed the nerve ultrastructure by electron microscopy. At P14, large caliber axons in control nerves were properly sorted from axon bundles, in a 1:1 relationship with SCs and overwhelmingly surrounded by myelin sheaths (Figure 4E). By contrast, some large caliber axons in sciatic nerves of age-matched *Rnf40^ΔSC^* mice were still found in mixed bundles with small caliber axons pointing to a mild sorting defect (Figure 4G). However, many large caliber axons had achieved a 1:1 relationship with SCs. These axons were fully surrounded by SCs, typically possessing 1–2 turns of SC membrane around them, but were frequently not myelinated. Such a phenotype is most consistent with an arrest at the promyelinating stage. The few axons with myelin usually exhibited thinner myelin sheaths compared to control. Structural abnormalities such as myelin outfoldings were rare and comparable in control and *Rnf40^ΔSC^* nerves. At later times such as P28 or P56, the myelination process had further progressed and reached a steady state in sciatic nerves of control mice (Figure 4F). Sciatic nerves of age-matched *Rnf40^ΔSC^* mice, however, did not substantially change in appearance as compared to P14. If anything, the percentage of unmyelinated large caliber axons had increased (Figure 4H). Upon closer inspection, some axons at P28 or later time points were no longer associated with a SC (Figure 4I). Some showed remnants of a basal lamina that pointed to the former presence of a SC (Figure 4J). Other axons were only partly wrapped by their associated SC or were surrounded by a SC with partly detached basal lamina (Figure 4K and L). Additionally, cells with a SC-like morphology were present in nerves that were not in contact with any axon (Figure 4M). We also found SCs that contained lipophilic droplet-like inclusions (Figure 4N). Similar inclusions were also observed in macrophages and perineurial cells (Figure 4O and P). There were also signs of degenerating axons (Figure 4Q).

Quantification of semi- and ultrathin sections confirmed the impression from visual inspection. Whereas almost all large caliber axons were successfully myelinated at P14 in controls, 37.1 ± 2.4% lacked a myelin sheath in *Rnf40^ΔSC^* nerves (Figure 4R). This proportion increased to 78.3 ± 1.3% at P56 arguing that a fraction of axons was never myelinated and that another fraction lost their myelin sheaths later on. Myelinated axons in *Rnf40^ΔSC^* nerves furthermore exhibited a higher g-ratio at all time points of analysis (Figure 4S). Whereas mean g-ratios in control sciatic nerves varied between values of 0.53 and 0.59, those in *Rnf40^ΔSC^* nerves had a mean value of 0.73 ± 0.01 at P14, which increased further to 0.84 ± 0.03 at P168. All axons above a diameter of 1 μm were affected by hypomyelination as obvious from scatter plots and binning analyses (Supplementary Figure S4E–L). As already judged from visual inspection, the number of large caliber axons was substantially reduced in sciatic nerves of *Rnf40^ΔSC^* mice as compared to age-matched controls from P28 onwards (Figure 4T). Immunohistochemistry of teased fibers with antibodies directed against Caspr and Na_v_1.6 visualized node and paranode in control fibers at P28 (Figure 4U). In contrast, fibers of *Rnf40^ΔSC^* nerves presented an irregular punctate Caspr signal and lacked detectable Na_v_1.6 staining without obvious correlation to their state of myelination (Figure 4V). In summary, we conclude that most SCs in *Rnf40^ΔSC^* nerves become arrested at the promyelinating stage. Those that eventually myelinate axons produce thin myelin sheaths, but are frequently unable to maintain their myelin. As a consequence, axons are un- or hypomyelinated, lack functional nodes of Ranvier, lose contact to SCs and eventually degenerate.

### Altered gene expression and H2Bub1 distribution in *Rnf40^ΔSC^* nerves

**Figure 5.**
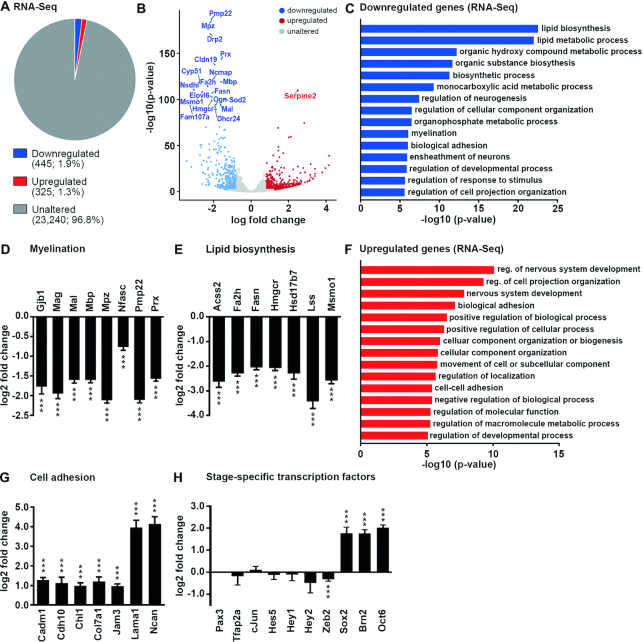
Altered gene expression in *Rnf40^ΔSC^* nerves. (**A**) Pie chart of expression data from RNA-Seq studies on sciatic nerves at P14 depicting genes with ≥1.75-fold downregulated (blue), ≥1.75-fold upregulated (red) and unchanged (gray) expression in *Rnf40^ΔSC^* nerves relative to controls. (**B**) Volcano plot showing overall changes in gene expression and the 20 most deregulated genes in *Rnf40^ΔSC^* nerves at P14. (**C**) GO analysis for biological processes enriched among genes downregulated in *Rnf40^ΔSC^* nerves. Processes are sorted by statistical significance. (**D**, **E**) Changes of expression for select genes associated with myelination (D) and lipid biosynthesis (E) according to RNA-Seq data (*n* = 3). (**F**) GO analysis for biological processes enriched among genes upregulated in *Rnf40^ΔSC^* nerves. (**G**, **H**) Changes of expression for select genes associated with cell adhesion (G) or coding for SC-specific transcription factors (H) according to RNA-Seq data (*n* = 3). Statistical significance was *P* ≤ 0.001 (***). Exact values are provided in Supplementary Tables.

To understand the molecular cause of hypomyelination, we isolated RNA from sciatic nerves of control and *Rnf40^ΔSC^* mice at P14 and performed RNA-Seq to compare the overall expression profiles between both genotypes. Samples from control and *Rnf40^ΔSC^* mice clustered separately according to principal component analysis (PCA) (Supplementary Figure S5A). A total of 445 genes exhibited a lower expression in *Rnf40^ΔSC^* nerves and 325 genes a higher expression (log_2_-fold change ≥0.8-fold; base mean ≥20, *P* ≤ 0.05; Figure 5A). Among the 20 most significantly deregulated genes, 19 were downregulated and only 1 upregulated (Figure 5B). Genes downregulated in *Rnf40^ΔSC^* nerves were associated with lipid biosynthesis, fatty acid metabolism, myelination and ensheathment by gene ontology (GO) analysis and gene set enrichment analysis (GSEA) (Figure 5C; Supplementary Figure S5B, C). Among others, these included genes that encode structural components of the myelin sheath such as *Gjb1, Mag, Mal, Mbp, Mpz, Nfasc, Pmp22* and *Prx*, as well as genes for essential lipid biosynthetic enzymes such as *Acss2, Fa2h, Fasn, Hmgcr, Hsd17b7, Lss* and *Msmo1* (Figure 5B, D and E). This correlates well with the strong hypomyelination observed in *Rnf40^ΔSC^* nerves.

In contrast, upregulated genes were preferentially associated with very general, uninformative terms or specific terms related to organization of cell projections, adhesion and movement of subcellular components (Figure 5F). Upregulated genes included those for cell adhesion molecules *Cadm1, Cdh10, Chl1, Col7a1, Jam3, Lama1* and *Ncan* (Figure 5G). Again, this corresponds well to the phenotype observed in *Rnf40^ΔSC^* nerves where most SCs were still in the promyelinating stage and established contact with their associated axon. Interestingly, there was no indication from GO analysis or GSEA that genes related to inflammation and immune response were substantially upregulated in *Rnf40^ΔSC^* nerves at P14 confirming that immune system activation is a delayed secondary response (Supplementary Figure S5D).

We have recently characterized a mouse mutant where early regulators of SC development are not properly shut off and cause a differentiation defect by their persistent expression (25). To address the potential relevance of this phenomenon in Rnf40-deficient SCs, we analyzed the expression of genes associated with transcriptional control of SC development. Detailed inspection revealed that most early regulators of SC development including *Pax3, Tfap2a, cJun, Hes5*, *Hey1, Hey2* and *Zeb2* did not exhibit substantial changes in their expression arguing that they remained appropriately shut off in Rnf40-deficient SCs (Figure 5H). In contrast, dramatic changes were observed for the later developmental regulators *Sox2, Brn2* (also known as *Pou3f2*), and *Oct6* (also known as *Pou3f1*) with 3.4- and 4-fold upregulation in *Rnf40^ΔSC^* nerves (Figure 5H).

**Figure 6.**
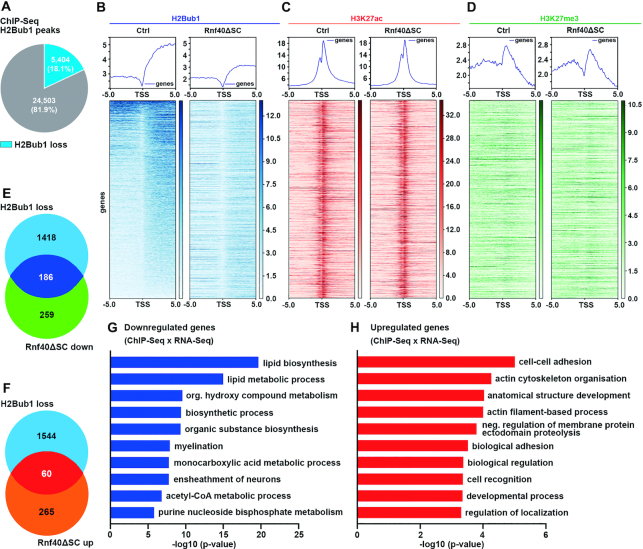
Altered H2Bub1 and other histone marks in *Rnf40^ΔSC^* nerves. (**A**) Pie chart depicting the total number of H2Bub1 peaks from ChIP-Seq in control sciatic nerves at P14 and the number of peaks lost in *Rnf40^ΔSC^* nerves (blue). (**B**–**D**) Plot profiles and heatmaps of H2Bub1 (B), H3K27ac (C) and H3K27me3 (D) ChIP-Seq signals in control (left panels) and *Rnf40^ΔSC^* (right panels) nerves ± 5 kb around the transcriptional start site (TSS) of genes associated with H2Bub1 peaks at P14. The order of genes from top to bottom is according to the strength of the H2Bub1 signal in the control. (**E**, **F**) Venn diagrams showing overlap of genes with decreased H2Bub1 peaks with genes exhibiting downregulated (E) or upregulated (F) expression in *Rnf40^ΔSC^* nerves. (**G**, **H**) GO analysis for biological processes linked to genes with decreased H2Bub1 peaks and downregulated (G) or upregulated (H) expression in *Rnf40^ΔSC^* nerves.

In a second approach, we analyzed H2Bub1 distribution throughout the genome in control and *Rnf40^ΔSC^* nerves by performing ChIP-Seq studies at P14 (for PCA and Volcano plots, see Supplementary Figure S6A, B). We detected a total of 29907 genomic regions that were marked by H2Bub1 in control nerves (Figure 6A). Approximately 18% of these regions exhibited reduced H2Bub1 occupancy in *Rnf40^ΔSC^* nerves. When genes associated with H2Bub1 peaks were identified and ordered in a heatmap according to their H2Bub1 content around the transcriptional start sites in control nerves, a preferential localization of H2Bub1 downstream of the TSS was observed (Figure 6B). Compared to controls, H2Bub1 signal intensity downstream of the TSS was substantially reduced in *Rnf40^ΔSC^* nerves both on a general and a gene-specific level as evident from plot profile and heatmap.

In chromatin from control nerves, most of the genes marked by H2Bub1 at the start of their coding region also displayed high levels of histone 3 acetylated at lysine 27 (H3K27ac) in the immediate vicinity of their TSS as a histone mark associated with active transcription (Figure 6C). In contrast, trimethylation of lysine 27 (H3K27me3) in histone 3 as a modification associated with a repressed state of transcription was not readily detected in these regions (Figure 6D). This argues that most genes that are marked by H2Bub1 are in a transcriptionally active state in control nerves. Analysis of these genes in chromatin from *Rnf40^ΔSC^* nerves revealed no global change in the overall distribution and intensities of H3K27ac and H3K27me3 marks (compare left and right heatmaps in Figure 6C and D). We therefore conclude that loss of Rnf40 and concurrently of the associated E3 ligase activity and the H2Bub1 mark did not lead to dramatic overall changes in the chromatin state.

The 5404 regions that had lost H2Bub1 in chromatin from *Rnf40^ΔSC^* nerves were assigned to 1604 genes. Comparison with our RNA-Seq data revealed that 186 of the genes with altered H2Bub1 signatures exhibited decreased expression in *Rnf40^ΔSC^* nerves as compared to controls (Figure 6E; listed in Supplementary Figure S6C). The number of genes with increased expression was approximately three times lower and amounted to 60 (Figure 6F; listed in Supplementary Figure S6D). The poor correlation between H2Bub1 loss and altered gene regulation was not unexpected, as previous studies had found Rnf40-dependent regulation and changed expression only for genes with moderate, but not with high H2Bub1 levels (4). This observation notwithstanding, the combined 246 genes are good candidates for direct target genes of Rnf40 in SCs. Genes downregulated in *Rnf40^ΔSC^* nerves were again primarily associated with lipid biosynthesis, fatty acid metabolism, myelination and axon ensheathment (Figure 6G), whereas upregulated genes had roles in cell adhesion, actin cytoskeleton organization, cell recognition and related terms (Figure 6H). Examples of downregulated genes with altered H2Bub1 occupancy include *Mbp, Mpz, Fads1* and *Fah2* (for IGV tracks see Supplementary Figure S7A–D). For *Sox2* and *Oct6*, H2Bub1 loss around the TSS correlated with an increased expression (for IGV tracks see Supplementary Figure S7E, F). We conclude from these findings that altered H2Bub1 occupancy led to positive as well as negative changes in gene expression.

### Molecular cause of hypomyelination in *Rnf40^ΔSC^* nerves

Intriguingly, Sox2 and Oct6 were among the genes that had lost H2Bub1 at their TSS and were upregulated in *Rnf40^ΔSC^* nerves. An Rnf40-dependent mechanism may therefore be involved in the repression of these two key transcription factors in SCs. As Sox2 is associated with the immature state and Oct6 with the promyelinating state, their continued expression could be an important, causally contributing factor to the SC defect in *Rnf40^ΔSC^* nerves.

The impact of a persistent Oct6 expression is difficult to address experimentally in vivo. Because of the requirement of Oct6 for regular progression of SC development (37,39), deletion would have to be experimentally induced immediately after the promyelinating stage in early differentiating SCs of *Rnf40^ΔSC^* mice and this is currently not feasible in mice with available genetic tools. However, the role of Sox2 expression is amenable to analysis as SC-specific *Sox2* deletion has only minor effects on SC development and developmental myelination (40). To investigate the contribution of continued Sox2 expression to the phenotypic defects in *Rnf40^ΔSC^* nerves, we generated mouse mutants in which Sox2 was deleted in addition to Rnf40 in SCs (Supplementary Figure S8C, G, K). By macroscopic inspection, *Sox2^ΔSC^ Rnf40^ΔSC^* mice exhibited a peripheral neuropathy with comparable severity to *Rnf40^ΔSC^* mice. Immunohistochemical analysis of sciatic nerves at P28 corroborated the phenotypic similarity. As quantified from DAPI stainings, *Sox2^ΔSC^ Rnf40^ΔSC^* nerves contained a slightly higher number of cells than controls as previously observed for *Rnf40^ΔSC^* nerves (Supplementary Figure S8A, E, I). Whereas the contribution of SCs to the sciatic nerve cell population was comparable to controls and *Rnf40^ΔSC^* nerves (Supplementary Figure S8B, F, J), the fraction of Oct6-positive SCs was increased to the same extent as in *Rnf40^ΔSC^* nerves (Supplementary Figure S8D, H, L). The number of Mbp- or Mpz-positive myelin sheaths and myelinating SCs remained dramatically reduced (Supplementary Figure S8M, N, P, Q, S, T), whereas Iba1-positive macrophages were similarly increased in *Sox2^ΔSC^ Rnf40^ΔSC^* as in *Rnf40^ΔSC^* nerves (Supplementary Figure S8O, R, U). Considering the lack of a rescue, continued Sox2 expression is not the major decisive determinant of the peripheral neuropathy in *Rnf40^ΔSC^* mice, but rather one of several contributing factors.

**Figure 7.**
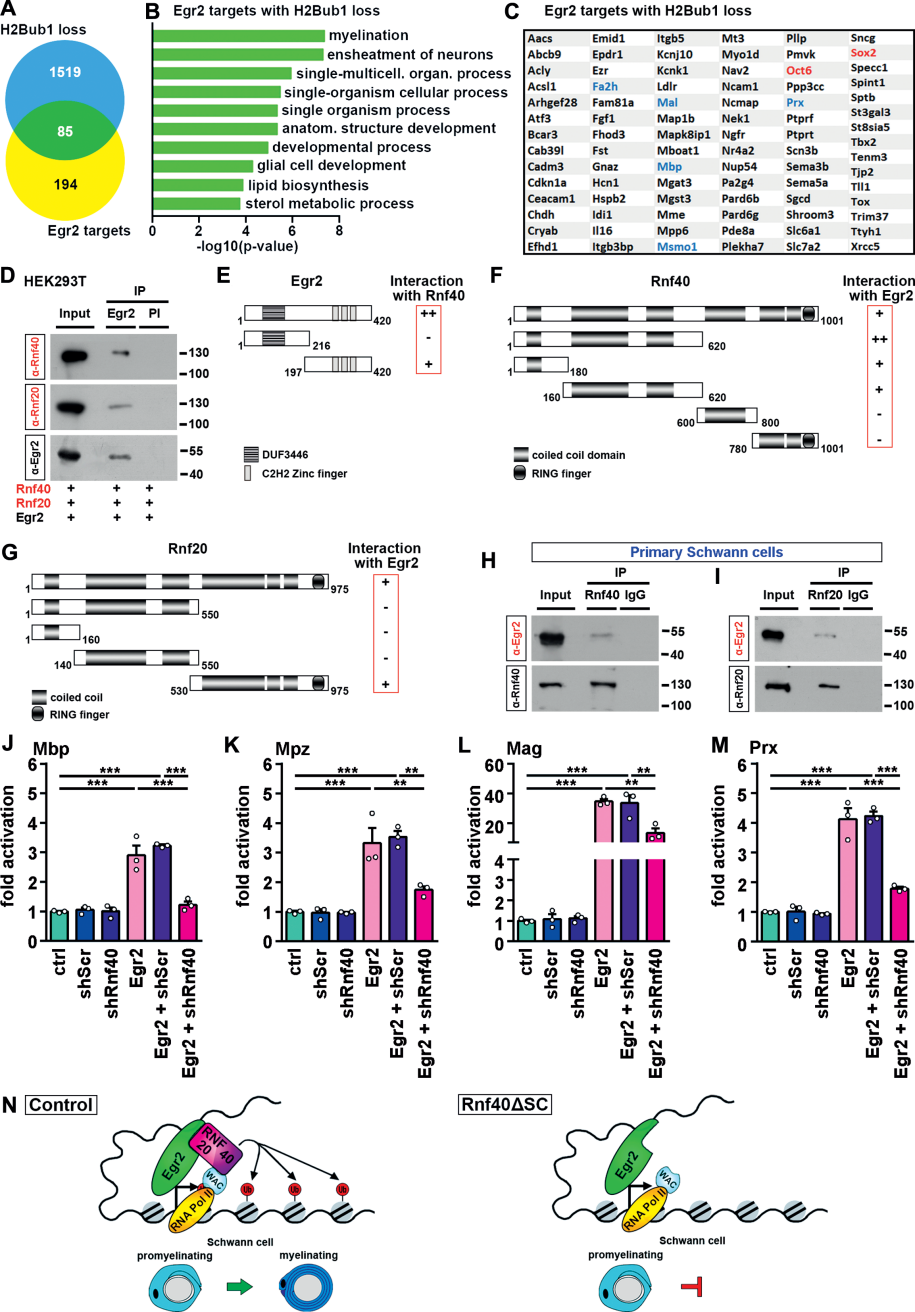
Physical and functional interaction of Egr2 and Rnf40/Rnf20. (**A**) Venn diagram depicting the overlap of Egr2 target genes with lost H2Bub1 marks in *Rnf40^ΔSC^* nerves. (**B**, **C**) GO analysis (B) and list (C) of Egr2 target genes with reduced H2Bub1 marks in *Rnf40^ΔSC^* nerves. (**D**) Co-immunoprecipitation (IP) of Rnf40 and Rnf20 with antibodies directed against Egr2 and pre-immune serum (PI) from extracts of HEK293T cells that were transfected with various expression plasmids as indicated below the panels. Western blot was used to detect precipitated Rnf40 (upper panel), Rnf20 (middle panel) and Egr2 (lower panel). Numbers on the right indicate position of co-electrophoresed size markers in kDa. (**E**) Scheme of Egr2 proteins and their ability to interact with myc-tagged Rnf40 in extracts from transfected HEK293T cells. DUF3446, domain of unknown function. (**F**) Scheme of myc-tagged Rnf40 proteins and their ability to interact with Egr2 in extracts from transfected HEK293T cells. (**G**) Scheme of HA-tagged Rnf20 proteins and their ability to interact with Egr2 in extracts from transfected HEK293T cells. (**H**, **I**) Co-immunoprecipitation (IP) of Egr2 with antibodies directed against Rnf40 (H), Rnf20 (I) and IgG control (H, I) from extracts of differentiating SC cultures. Western blot was used to detect precipitated Egr2 (upper panels), Rnf40 (lower panel, H) and Rnf20 (lower panel, I). Experiments were repeated three times. Uncropped western blots for D–I are presented as Supplementary Figures S9 and S10. (**J**–**M**) Determination of endogenous expression levels of *Mbp* (J), *Mpz* (K), *Mag* (L) and *Prx* (M) by qRT-PCR in Neuro2a cells transfected with various combinations of expression plasmids for Egr2, Rnf40-specific shRNA (shRnf40), scrambled shRNA (shScr) or various combinations of these as indicated below the lanes after FACS. Transcript levels in cells transfected with empty expression vectors (ctrl) were set to 1 and all other values were expressed in relation to it (*n* = 3; mean values ± SEM). Statistical significance was determined by one-way ANOVA with Bonferroni's multiple comparisons test (*P ≤ 0.05; **P ≤ 0.01; ***P ≤ 0.001). Exact values are provided in the Supplementary Tables. (**N**) Proposed model for the action of the Rnf40/Rnf20 E3 ligase and H2Bub1 modification in SC development.

Both *Sox2* and *Oct6* have previously been reported to be targets of Egr2 repression (36,41,42). This opens the possibility that persistent expression of Sox2 and Oct6 is a consequence of compromised Egr2 function in the absence of Rnf40/Rnf20 E3 ligase. To analyze whether there is a more general link between Egr2 function and Rnf40/Rnf20 activity, we compiled a list of 279 direct Egr2 target genes from published RNA-Seq and ChIP-Seq studies (30,31) and compared this list with genes that had reduced H2Bub1 marks in *Rnf40^ΔSC^* nerves. Indeed, we found that 85 of the 279 Egr2 target genes displayed a loss of H2Bub1 downstream of their TSS (Figure 7A). These included genes whose products code for myelin components such as *Mbp* or are central in lipid metabolism as evident from GO analysis (Figure 7B and C). Interestingly, neither myelin nor lipid biosynthesis associated genes were significantly enriched among the 194 Egr2 target genes with unaltered H2Bub1 status (Supplementary Figure S7G). Egr2 target genes with H2Bub1 loss exhibited a similarly high overlap with Sox10 target genes (43) than Egr2 target genes without such loss (74.1% and 78.3%, respectively) arguing against an influence of Sox10 on H2B monoubiquitination. Similar results were also obtained when comparing Egr2 and Nab1/2 target genes in SCs (36).

Considering that genes with altered H2Bub1 in *Rnf40^ΔSC^* nerves are substantially enriched among Egr2 target genes (30% as compared to 7% for all genes), we postulated a functional link between Egr2 and the Rnf40/Rnf20 E3 ligase. We hypothesized that Egr2 might physically recruit the H2B ubiquitin ligase complex to some of its target genes as a precondition for ensuring the expression levels required in myelinating SCs. Therefore, we investigated whether Egr2 physically interacts with Rnf40 or Rnf20 by co-immunoprecipitation experiments. For this purpose, we first used protein extracts from HEK293T cells that had been transfected with Egr2, Rnf40 or Rnf20 expression plasmids. Antibodies directed against Egr2 were able to co-precipitate Rnf40 and Rnf20 from extracts that contained overexpressed proteins (Figure 7D, Supplementary Figure S9A).

To further prove specificity and map the regions involved in the interactions, we generated truncated versions of Rnf40, Rnf20, and Egr2 proteins and tested them for their ability to co-precipitate (Figure 7E–G). Wherever possible, we tried to keep protein domains (as determined by the COILS prediction program in the ExPASy Bioinformatics Resource Portal) intact. In case of Rnf40, myc-tagged domains were overexpressed and tested for co-precipitation by anti-Egr2 antibodies (Figure 7F, Supplementary Figure S9B–F). Whereas a domain containing the first 620 amino acids of Rnf40 was readily detected in the precipitate, two carboxyterminal fragments spanning amino acids 600–800 and 780–1001 were not co-precipitated, arguing that the interaction of Rnf40 with Egr2 is mediated by its aminoterminal half. When further divided, both the first 180 amino acids and a region spanning amino acids 160–620 interacted with Egr2, albeit at lower efficiency. Considering that these two domains overlap by only 20 amino acids it is very likely that both parts are independently able to bind to Egr2 and are jointly required for full binding affinity. In case of Rnf20, interaction with Egr2 was mapped using HA-tagged protein fragments (Figure 7G, Supplementary Figure S10A–D). Intriguingly, only the carboxyterminal half of the protein spanning amino acids 530–975 interacted with Egr2, whereas none of the aminoterminal parts, spanning amino acids 1–550, 1–160 or 140–550 of Rnf20 showed this property. Therefore, both subunits of the E3 ligase seemed to independently interact with Egr2 via different domains. It deserves to be mentioned that interaction with Rnf40 was previously mapped to the aminoterminal half of Rnf20 (2) indicating that different parts of the protein are involved in interaction with Rnf40 and Egr2, respectively. It is also unlikely that Egr2 binding disturbs the interaction of either Rnf40 or Rnf20 with its functionally essential interaction partner Wac, which occurs via their third coiled-coil domain (44). We also tested several parts of Egr2 for their ability to interact with Rnf40 in co-immunoprecipitations with anti-myc antibodies from extracts containing aminoterminal myc-tagged Rnf40 (Figure 7E, Supplementary Figure S10E, F). In case of Egr2, interaction was mapped to a fragment that spans amino acids 197–420 and corresponds to the carboxyterminal zinc finger-containing half of the protein. In contrast, the aminoterminal half of Egr2 from amino acids 1 to 216 did not interact.

To investigate whether the physical interaction between Egr2 and the Rnf40/Rnf20 complex is also relevant in PNS glia, we repeated the co-immunoprecipitation experiments with extracts from primary rat SCs that had been treated for 3 days with dibutyryl-cAMP, neuregulin and insulin to initiate differentiation and induce Egr2 expression. Endogenous Egr2 was successfully co-precipitated from SC extracts with both anti-Rnf40 and anti-Rnf20 antibodies (Figure 7H, I, Supplementary Figure S10G, H). In summary, these results strongly point to a specific interaction between Egr2 and the Rnf40/Rnf20 complex and confirm its existence in SCs.

Given the fact that Egr2 and the Rnf40/Rnf20 complex share target genes in differentiating SCs and physically interact, we finally looked for evidence of functional interaction. For this purpose, we analyzed the ability of ectopic Egr2 to induce the expression of its target genes in Neuro2a neuroblastoma cells with varying levels of Rnf40/Rnf20 E3 ligase activity. We used Neuro2a cells because primary SCs proved difficult to transfect with sufficient efficiency and were variable between preparations. Neuro2a cells on the other hand can be transfected at high rates and are homogeneous. They also share a common neural crest origin with SCs. Despite the close ontogenetic relationship, we were unable to test the repressive effect of Egr2 on *Sox2* or *Oct6* expression as these genes are not transcribed in Neuro2a cells as determined by qRT-PCR. Furthermore, transcription of genes linked to lipid biosynthesis was already so high in Neuro2a cells that it could not be further boosted by ectopic Egr2. Thus among Egr2 target genes, we chose myelin genes such as *Mbp, Mpz, Mag* and *Prx* for further analysis, because they were expressed at low basal levels in these cells and exhibited increased expression after transfection with Egr2 expression plasmids (Figure 7J–M). When Neuro2a cells were transfected with a shRNA directed against Rnf40 to knockdown Rnf40/Rnf20 E3 ligase activity or a scrambled control, low level myelin gene expression was unaffected (Figure 7J–M). However, when Neuro2a cells were transfected with expression plasmids for Egr2 and Rnf40-specific shRNA, the Egr2-dependent increase in myelin gene expression was severely blunted. Such an effect was not observed when the Rnf40-specific shRNA was replaced by a scrambled control. These data argue that Egr2-dependent activation of myelin genes requires the presence of functional Rnf40/Rnf20 E3 ligase.

## DISCUSSION

We here show that SC-specific deletion of Rnf40 and the resulting loss of H2Bub1 led to severe hypomyelination that secondarily triggers an inflammatory response and a progressive loss of axons and thereby interfered with peripheral nerve function. Apart from a study on the heart where Rnf40 and H2Bub1 impact development via epigenetic control of cilia motility (45), this is to our knowledge the only study where the role of H2B monoubiquitination and the responsible E3 ligase has been investigated during ontogenetic development. We also like to point out that apart from studies on histone deacetylases (46,47), very few data exist on the role of specific histone modifications in SCs.

In line with previous reports on cellular systems (12,14,16,21), we found evidence for a role of H2Bub1 in determining cell-specific gene expression and differentiation. In the absence of Rnf40 and H2Bub1, we observed only a mild axon sorting defect. Many SCs progressed on schedule into the promyelinating stage, but then failed to establish high level expression of genes that code for myelin proteins and proteins involved in lipid biosynthesis as a precondition for myelin sheath formation. As a consequence, many Schwann cells arrested their differentiation at the promyelinating stage and failed to undergo differentiation to mature, myelinating SCs. Some cells produced myelin. However, this myelin was too thin, could not be maintained over longer periods and disappeared in older animals resulting in progressive axonal degeneration.

As in other cell types, H2Bub1 is enriched in numerous regions throughout the SC genome. Therefore, it was surprising that its loss did not lead to global changes in SC gene expression. Instead, <250 genes exhibited significant alterations in both H2Bub1 occupancy and gene expression. Of these affected target genes, more were expressed at lower levels in the absence of H2Bub1 than at higher levels and the level of downregulation was in general stronger than the level of upregulation. These results are consistent with previous work in Rnf40-deficient mouse fibroblasts and support the notion that H2Bub1 occupancy is predominantly associated with an active state of transcription, but influences transcription only for a select subset of H2Bub1-occupied genes (4).

In Rnf40-deficient SCs many genes with reduced expression after H2Bub1 loss represent genes that code for key myelin proteins or are involved in the production of the membrane lipids. These genes must be transcribed at very high levels during myelination so that dependency of their expression on H2Bub1 may result from the ability of H2Bub1 to boost transcription, for instance by increasing processivity of RNA polymerase II and recruiting transcription elongation complexes (3,48).

However, we also found genes with increased expression after H2Bub1 loss in Schwann cells, suggesting that H2Bub1 occupancy may sometimes suppress gene expression in a context-dependent and tissue-specific manner. Again, this is in good agreement with previous studies (5,17). One prominent gene that is normally repressed in Schwann cells by H2Bub1 is Sox2, a transcription factor closely associated with the immature state (36). Prolonged Sox2 expression in SCs has been shown to interfere with myelination and increase macrophage numbers in the peripheral nerve of transgenic animals (40). Phenotypic defects in *Rnf40^ΔSC^* mice may therefore be a consequence of the continued Sox2 expression in Rnf40-deficient SCs. However, we show in our study that correction of the aberrant Sox2 expression alone does not rescue the phenotype in our mouse mutants.

Another gene under H2Bub1 repression is Oct6, a transcription regulator of the promyelinating stage (37). In the adult SC, Oct6 needs to be shut off as its continued expression interferes with myelin homeostasis and maintenance of a mature state (49). We currently assume that both the persisting expression of Sox2 and Oct6 as well as the shortage of myelin proteins and lipids impede the proper generation of myelin sheaths. Thus, SCs are effectively stalled in the promyelinating stage or an early abnormal myelinating stage and prevented from reaching a fully mature homeostatic state.

Intriguingly, a substantial number of the genes that are affected in their expression by H2Bub1 loss have previously been identified as target genes of Egr2 (50). This transcription factor represents the central regulator of SC differentiation and peripheral myelination and is both involved in the downregulation of Sox2 and Oct6 as well as the upregulation of genes coding for myelin and lipid metabolic genes (36,41,51,52). Importantly, our study identified multiple direct interactions between Egr2 and the subunits of the H2B monoubiquitinating E3 ligase, and even showed that the Egr2-dependent induction of myelin genes in a heterologous cell line depends on the presence of Rnf40. To explain Rnf40 function in SCs, we therefore propose the following mode of action (Figure 7N). Once Egr2 is induced, it recruits the Rnf40/Rnf20 E3 ligase to a subset of its target genes resulting in H2B monoubiqitination around the transcriptional start site. This monoubiquitination facilitates chromatin state transitions and thereby ensures that several myelin and lipid metabolic genes are transcribed at sufficiently high levels to allow formation of myelin sheaths and their maintenance. Egr2-driven local Rnf40 recruitment and H2B monoubiquitination also leads to downregulation of Sox2 and Oct6 and thereby helps Egr2 to establish homeostatic control and a stable mature state in SCs. This mechanism would also predict that loss of H2B monoubiquitination in SCs may be instrumental in driving dedifferentiation and reprogramming into repair SCs under conditions where remyelination is required such as after nerve injury (53), and may thus represent a therapeutic target to enhance remyelination efficiency.

## DATA AVAILABILITY

All data generated or analyzed during this study are included in this published article and its supplementary information or were deposited in GEO under accession numbers GSE146646 [https://www.ncbi.nlm.nih.gov/geo/query/acc.cgi?acc=GSE146646], GSE146629 [https://www.ncbi.nlm.nih.gov/geo/query/acc.cgi?acc=GSE146629] and GSE146645 [https://www.ncbi.nlm.nih.gov/geo/query/acc.cgi?acc=GSE146645].

## Supplementary Material

gkaa606_Supplemental_File
