# Supplementary material for: Egr2-guided histone H2B monoubiquitination is required for peripheral nervous system myelination
Source: Nucleic Acids Res. 2020 Jul 16;48(16):8959–76. doi: 10.1093/nar/gkaa606 (PMC7498331; doi:10.1093/nar/gkaa606)
Supplement: gkaa606_Supplemental_File [file gkaa606_supplemental_file.pdf]

Egr2-guided histone H2B monoubiquitination is required for peripheral nervous system myelination

Hannah M. Wüst[1], Amélie Wegener[1], Franziska Fröb[1], Anna C. Hartwig[1], Florian Wegwitz[2], Vijayalakshmi Kari[2], Margit Schimmel[3], Ernst. R. Tamm[3], Steven A. Johnsen[2,4], Michael Wegner[1*], Elisabeth Sock[1*]

Supplementary Data

SUPPLEMENTARY FIGURES

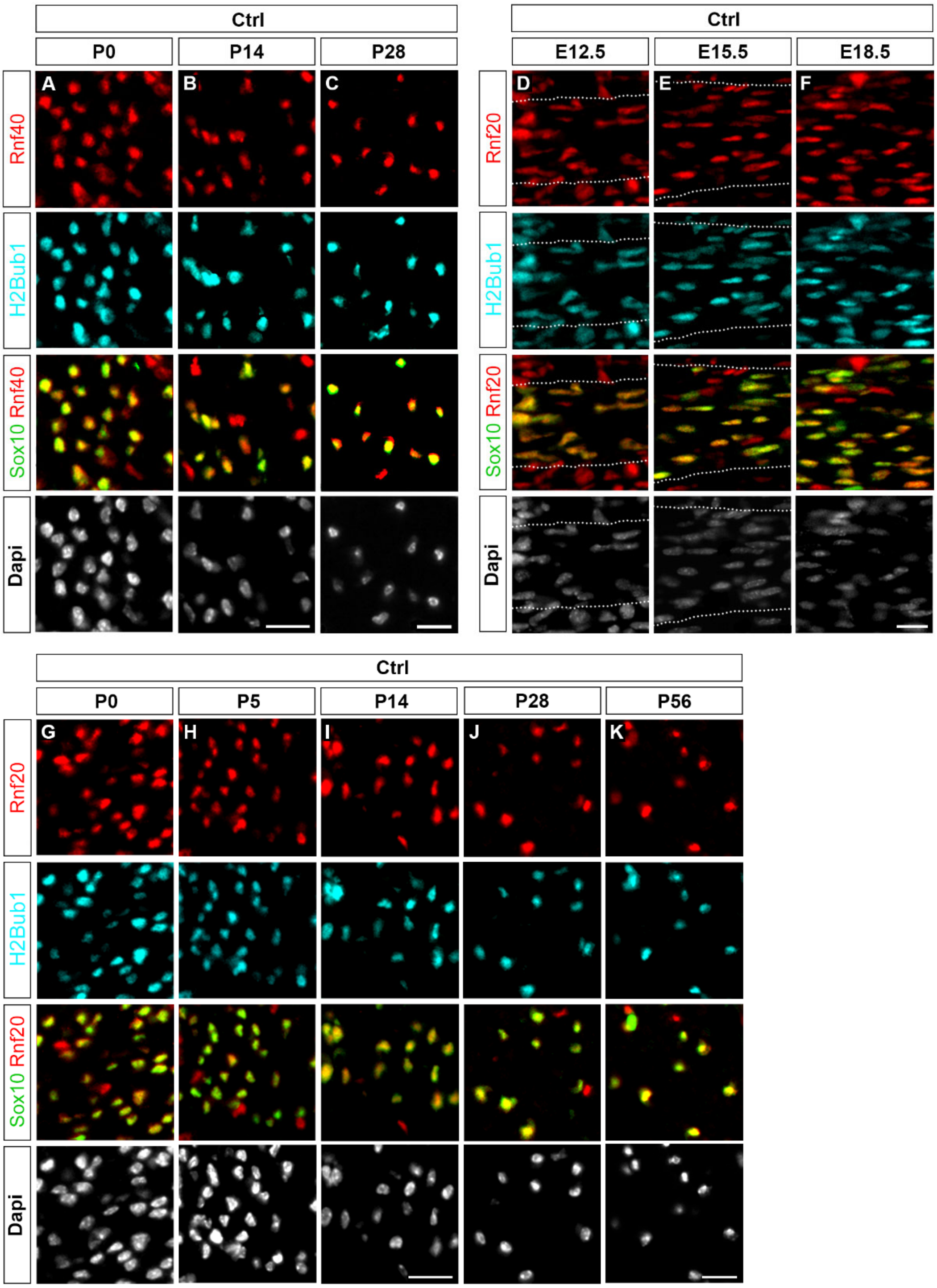

Supplementary Figure S1: Expression of Rnf40 and Rnf20 in SCs. (**A-C**) Temporal occurrence of Rnf40 in SCs of sciatic nerves from control mice at P0 (A), P14 (B) and P28 (C) as determined by co-immunofluorescence studies with antibodies against Rnf40 (red), H2bub1 (cyan) and Sox10 (green). (**D-K**) Temporal occurrence of Rnf20 in SCs of spinal (D-F) and sciatic (G-K) nerves from control mice at E12.5 (D), E15.5 (E), E18.5 (F), P0 (G), P5 (H), P14 (I), P28 (J) and P56 (K) as determined by co-immunofluorescence studies with antibodies against Rnf20 (red), H2bub1 (cyan) and Sox10 (green). Scale bars: 15µm.

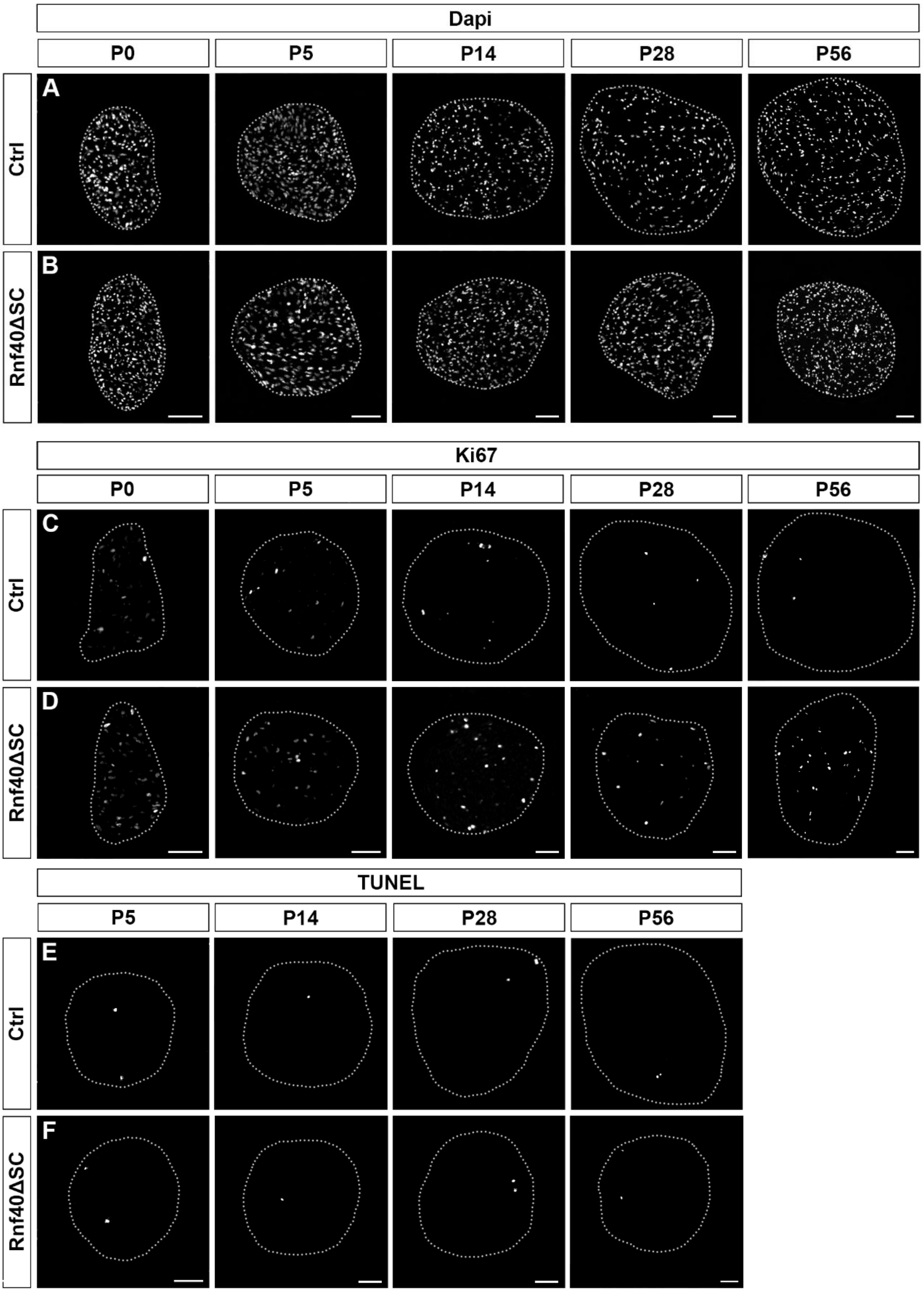

Supplementary Figure S2: Sciatic nerve cells in *Rnf40*$^{\Delta SC}$ mice. (**A,B**) DAPI-staining of sciatic nerve sections of control (A) and *Rnf40*$^{\Delta SC}$ (B) mice at P0, P5, P14, P28 and P56. (**C,D**) Immunohistochemical stainings of sciatic nerve sections of control (C) and *Rnf40*$^{\Delta SC}$ (D) mice from P0 to P56 with antibodies directed against Ki67. (**E,F**) TUNEL of sciatic nerve sections of control (E) and *Rnf40*$^{\Delta SC}$ (F) mice from P5 to P56. Sections were placed on a black background and are surrounded by a dotted line. Scale bars: 50µm.

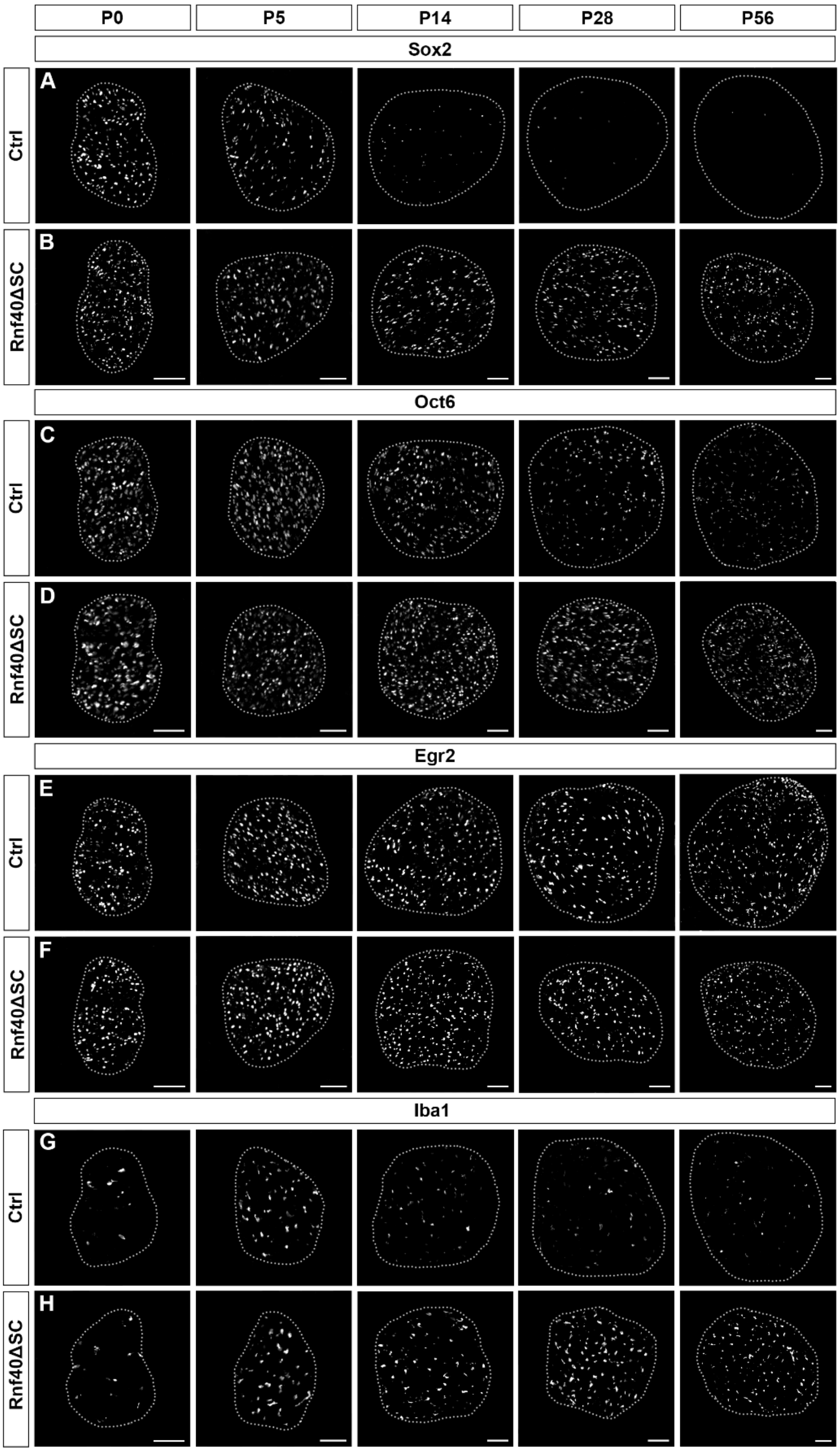

Supplementary Figure S3: Expression of cell and stage marker proteins in *Rnf40^{ΔSC}* mice. (**A-H**) Immunohistochemical stainings of sciatic nerve sections of control (A,C,E,G) and *Rnf40^{ΔSC}* (B,D,F,H) mice at P0, P5, P14, P28 and P56 with antibodies directed against Sox2 (A,B), Oct6 (C,D), Egr2 (E,F) and Iba1 (G,H). Sections were placed on a black background and are surrounded by a dotted line. Scale bars: 50µm.

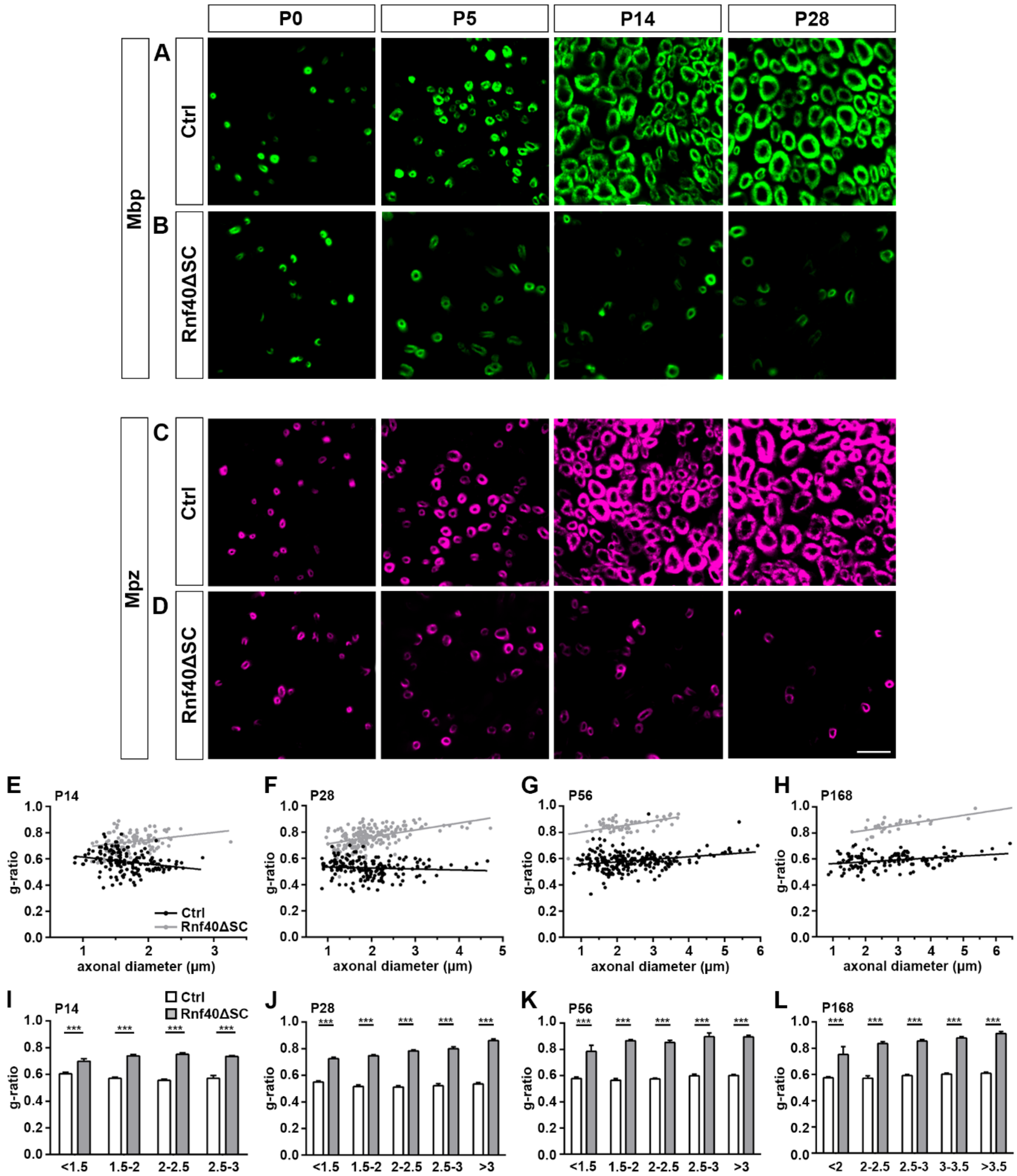

Supplementary Figure S4: Expression of myelin proteins in *Rnf40*$^{\Delta SC}$ mice. (**A-D**) Immunohistochemical stainings of sciatic nerve sections of control (A,C) and *Rnf40*$^{\Delta SC}$ (B,D) mice at P0, P5, P14 and P28 with antibodies directed against Mbp (A,B) and Mpz (C,D). Scale bar: 10µm. (**E-L**) Determination of single g-ratios (scatter plots in E-H) and mean g-ratio ± SEM in relation to axon size after binning of axons according to their diameter (in µm) (I-L) from ultrathin sciatic nerve sections of control (black dots and white bars) and *Rnf40*$^{\Delta SC}$ (gray dots and bars) mice at P14 (E,I), P28 (F,J), P56 (G,K) and P168 (H,L) using n = 100-300 axons for each age. Statistical significance was determined by unpaired two-tailed Student's t-test (*, P ≤0.05; **, P ≤0.01; ***, P ≤0.001). Exact values are provided in the Supplementary Tables.

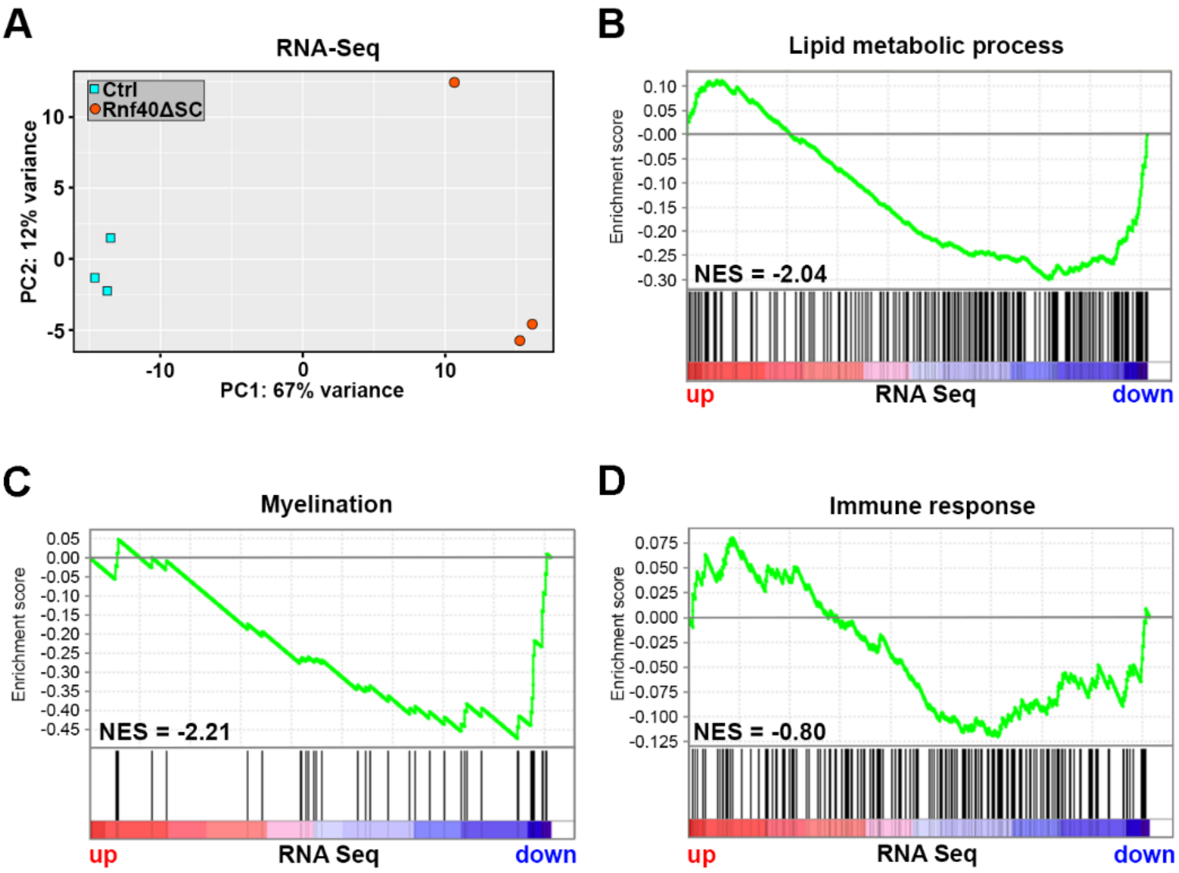

Supplementary Figure S5: RNA-Seq data from sciatic nerves of control and *Rnf40*$^{\Delta SC}$ mice. (**A**) PCA plot of RNA-Seq samples from sciatic nerves of control (cyan squares) and *Rnf40*$^{\Delta SC}$ (red dots) mice at P14. (**B-D**) Gene set enrichment plots from GSEA of RNA-Seq data with the following gene sets: lipid metabolic process (B), myelination (C) and immune response (D). The normalized enrichment score (NES) is shown in the lower left corner of each plot.

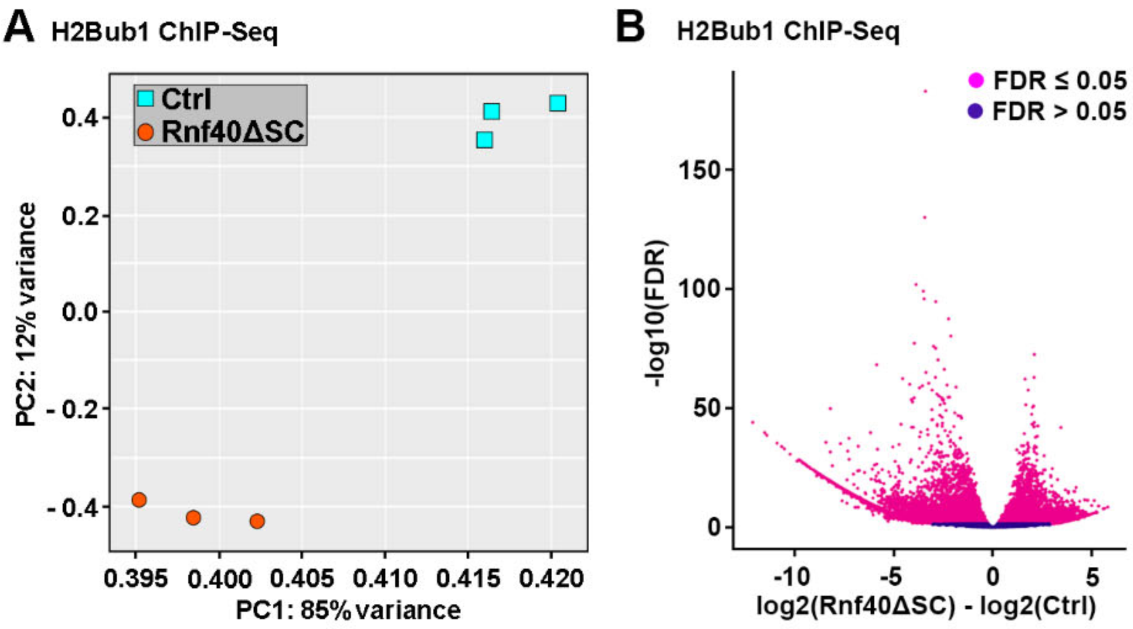

C Downregulated genes (ChIP-Seq)

0610007P14Rik	Adap1	Chp2	Eda	Fasn	Hspa4l	Luzp2	Myo1d	Plekhb1	Sc5d	Snx7
4932411E22Rik	Adcy1	Cldn19	Efcc1	Fdft1	Hspb2	Mag	Ncmap	Plxna3	Sccpdh	Sorbs1
5930403L14Rik	Agl	Clip3	Efhd1	Fdps	Ifit2	Mal	Nek1	Plxnb1	Scn7a	Sptb
B230206H07Rik	Ak5	Cmtm5	Ell2	Fgf1	Il16	Mapk8ip1	Ngfr	Plxnb3	Sema3b	Srpk3
D430019H16Rik	Ank3	Col23a1	Elovl6	Fhl3	Insig1	Matn2	Nkain2	Pmvk	Sema4g	Syna
D630003M21Rik	Ankrd13b	Col28a1	Elovl7	Frmd3	Itgb4	Matn4	Nln	Polr3g	Sept3	Syt11
D630045J12Rik	Anln	Ctnna3	Emid1	Fscn1	Kazn	Mboat1	Nr4a2	Prkcq	Serpinb6a	Tkt
Aacs	Apc2	Cyp2j6	Endod1	Gal3st1	Kcnk1	Mbp	Nrn1	Prrg1	Setd8	Tll1
Abca5	Arhgap19	Cyp39a1	Epdr1	Gas2l3	Kcnmb4	Megf6	Nsdhl	Prrg3	Sgk3	Tmem38a
Abca7	Art3	Cyp51	Fa2h	Gbe1	Kif19a	Mfsd2a	Nsmf	Prx	Shc4	Tmem62
Acaca	Aspa	Ddn	Faah	Gpd1	Ldlr	Mgst3	Ntrk3	Pter	Shpk	Tmod2
Acat2	Asrgl1	Deptor	Fads1	Gpr155	Lgi1	Mme	Olfml2b	Ptprt	Slc22a23	Trp53cor1
Acly	Cadm4	Dhcr24	Fads2	Gpr37l1	Lgi4	Mmp15	Optn	Rasgef1c	Slc25a23	Ugt8a
Acsl1	Cd59a	Dhcr7	Fam178b	H2-DMa	Lhpp	Mpz	Pank1	Reep6	Slc41a2	Vash1
Acsl3	Cdh19	Drp2	Fam184a	Habp4	Limch1	Msmo1	Pcsk6	Rimklb	Slc7a2	Vat1l
Acss2	Chdh	Dusp15	Fam26e	Hepacam	Lrrk2	Mt3	Pla2g16	Rnf157	Snca	Zc4h2
Adam22	Chn2	Ecscr	Fam83f	Hsd17b7	Lss	Mvk	Plekha4	Satb1	Sncg	

D Upregulated genes (ChIP-Seq)

2610035D17Rik	Cadm1	Csmd1	Fhod3	Hr	Megf10	Ncam1	Ptpn3	Syn3	Trpm3
Abcg1	Cdc42ep3	Cttnbp2	Foxd3	Igsf11	Mpzl1	Nipal1	Rnf165	Tbx2	Tspan13
Abtb2	Cdh6	Dock2	Gcnt1	Jam3	Mycn	Nr2f1	Serpine2	Tex9	Ust
Asxl3	Chl1	Elmo1	Gnai1	Kcnj10	Nav1	Pfas	Sox2	Timp3	Zfhx4
Bzw2	Chsy3	Ezr	Gpr137b	Lmo1	Nav2	Oct6	Spint1	Tmtc2	Zfp612
Cacna1d	Coro2b	Fam181b	Hcn1	Lrrc4b	Ncald	Ppp1r1b	Srgap1	Tnik	Zswim5

Supplementary Figure S6: ChIP-Seq data from sciatic nerves of control and *Rnf40*$^{\Delta SC}$ mice. (**A,B**) PCA (A) and Volcano (B) plots of ChIP-Seq samples from sciatic nerves of control (cyan squares) and *Rnf40*$^{\Delta SC}$ (red dots) mice at P14. (**C,D**) Lists of genes that exhibit decreased H2Bub1 peaks and downregulated (C) or upregulated (D) expression in *Rnf40*$^{\Delta SC}$ sciatic nerves at P14.

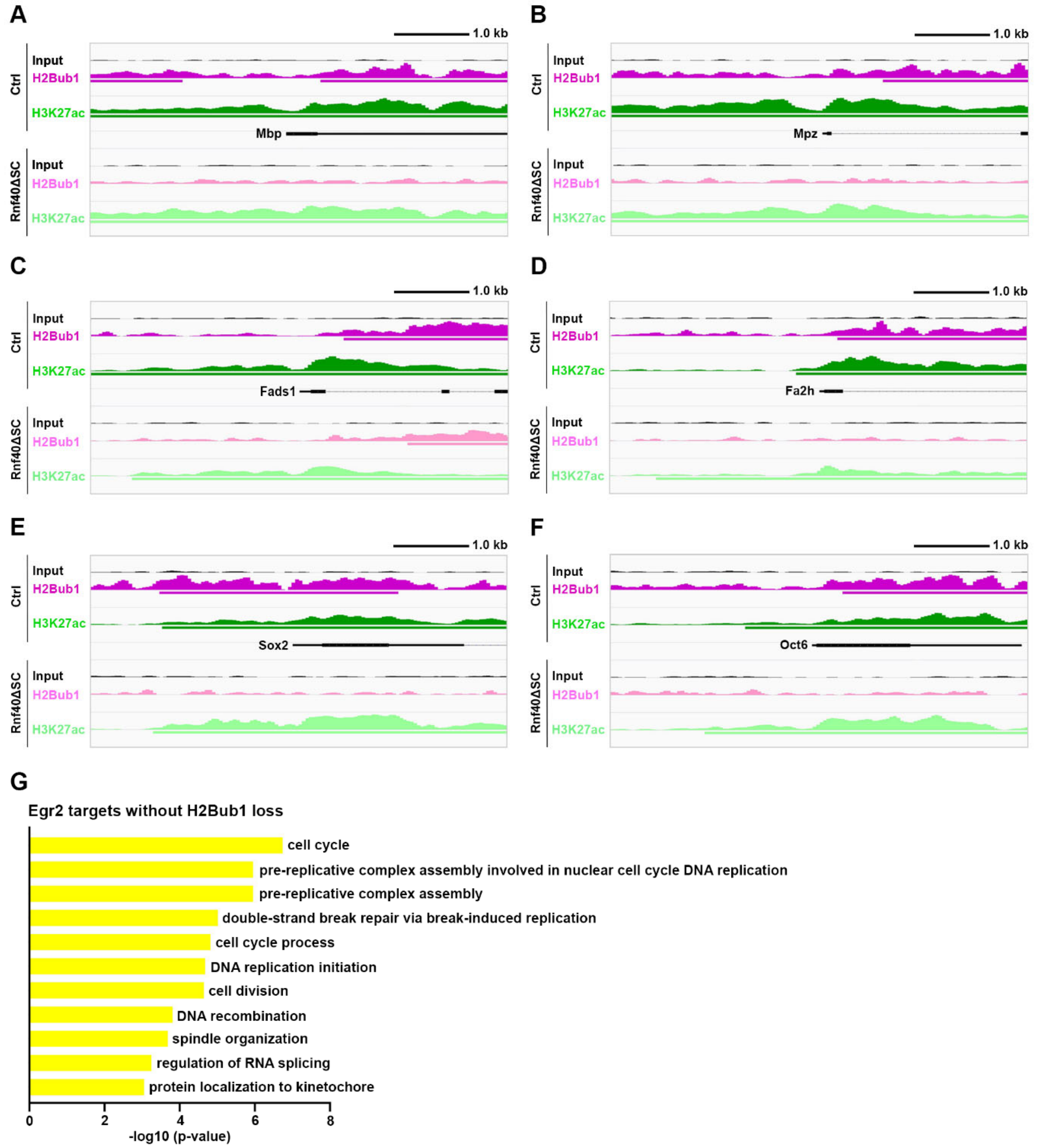

Supplementary Figure S7: Analysis of histone modifications around the TSS of select genes and Egr2 targets without H2Bub1 alterations. (**A-F**) Selected IGV tracks showing H2Bub1 and H3K27ac distribution around the TSS of *Mbp* (A)*, Mpz* (B)*, Fads1* (C)*, Fah2* (D)*, Sox2* (E) and *Oct6* (F) genes in nerves of control (upper part, dark colors) and *Rnf40*$^{\Delta SC}$ (lower part, light colors) as determined in ChIP-Seq experiments relative to input. Direction of transcription is from left to right. Transcripts are marked by a black line with line thickness increasing from intronic regions via exons to translated regions. Scale bar: 1 kb. (**G**) GO analysis of Egr2 target genes with unchanged H2Bub1 status in *Rnf40*$^{\Delta SC}$ nerves.

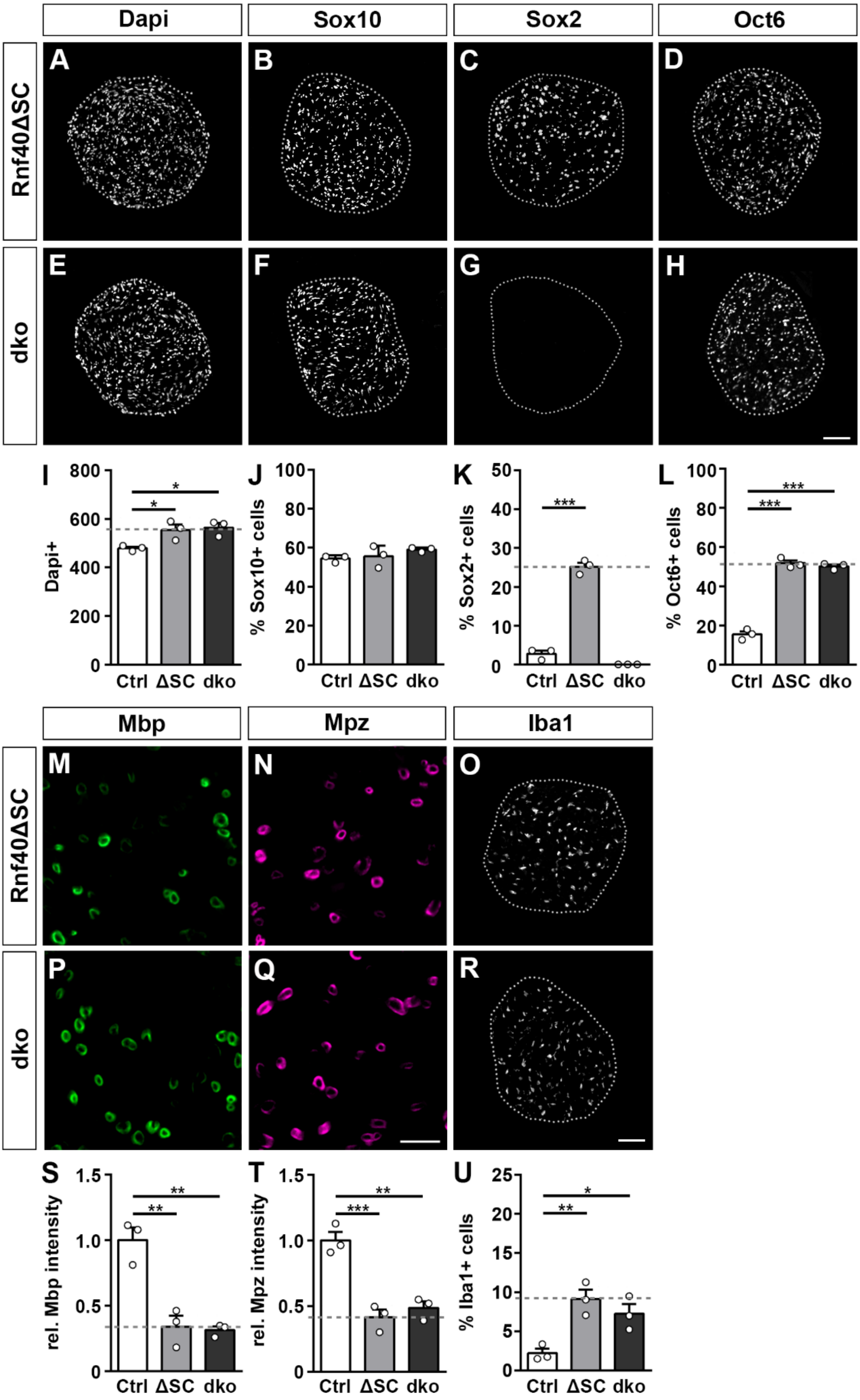
Dapi
Sox10
Sox2
Oct6
Rnf40ΔSC
dko
A
B
C
D
E
F
G
H
I
J
K
L
Dapi+
% Sox10+ cells
% Sox2+ cells
% Oct6+ cells
Ctrl ΔSC dko
Mbp
Mpz
Iba1
M
N
O
P
Q
R
S
T
U
rel. Mbp intensity
rel. Mpz intensity
% Iba1+ cells

Supplementary Figure S8: Sciatic nerve comparison in *Rnf40*$^{\Delta SC}$ and *Sox2*$^{\Delta SC}$ *Rnf40*$^{\Delta SC}$ mice. (**A-H**) Stainings of sciatic nerve sections of *Rnf40*$^{\Delta SC}$ (A-D) and *Sox2*$^{\Delta SC}$ *Rnf40*$^{\Delta SC}$ (dko) (E-H) mice at P28 with DAPI (A,E) and antibodies directed against Sox10 (B,F), Sox2 (C,G) and Oct6 (D,H). (**I-L**) Quantification of the number of total cells (I), the relative contribution of Sox10-positive SCs to the overall cell population (J) and the percentage of Sox2-positive immature (K) and Oct6-positive promyelinating (L) SCs among all SCs in sciatic nerve sections of control (Ctrl, white bars), *Rnf40*$^{\Delta SC}$ (ΔSC; light gray bars) and and *Sox2*$^{\Delta SC}$ *Rnf40*$^{\Delta SC}$ (dark gray bars) mice (n = 3; mean values ± SEM). (**M-R**) Stainings of sciatic nerve sections of *Rnf40*$^{\Delta SC}$ (M-O) and *Sox2*$^{\Delta SC}$ *Rnf40*$^{\Delta SC}$ (P-R) mice at P28 with antibodies directed against Mbp (M,P), Mpz (N,Q) and Iba1 (O,R). (**S-U**) Comparison of Mbp (S) and Mpz (T) signal intensities and the relative contribution of Iba1-positive macrophages to the overall cell population (U) in sciatic nerve sections of control, *Rnf40*$^{\Delta SC}$ and *Sox2*$^{\Delta SC}$ *Rnf40*$^{\Delta SC}$ mice at P28 by quantification of sections stained with respective antibodies (n = 3; mean values ± SEM). Scale bars: 10µm in Q, 50µm in H,R. Statistical significance was determined by One-way ANOVA with Bonferroni's multiple comparisons test (*, P ≤0.05; **, P ≤0.01; ***, P ≤0.001). Exact values are provided in the Supplementary Tables.

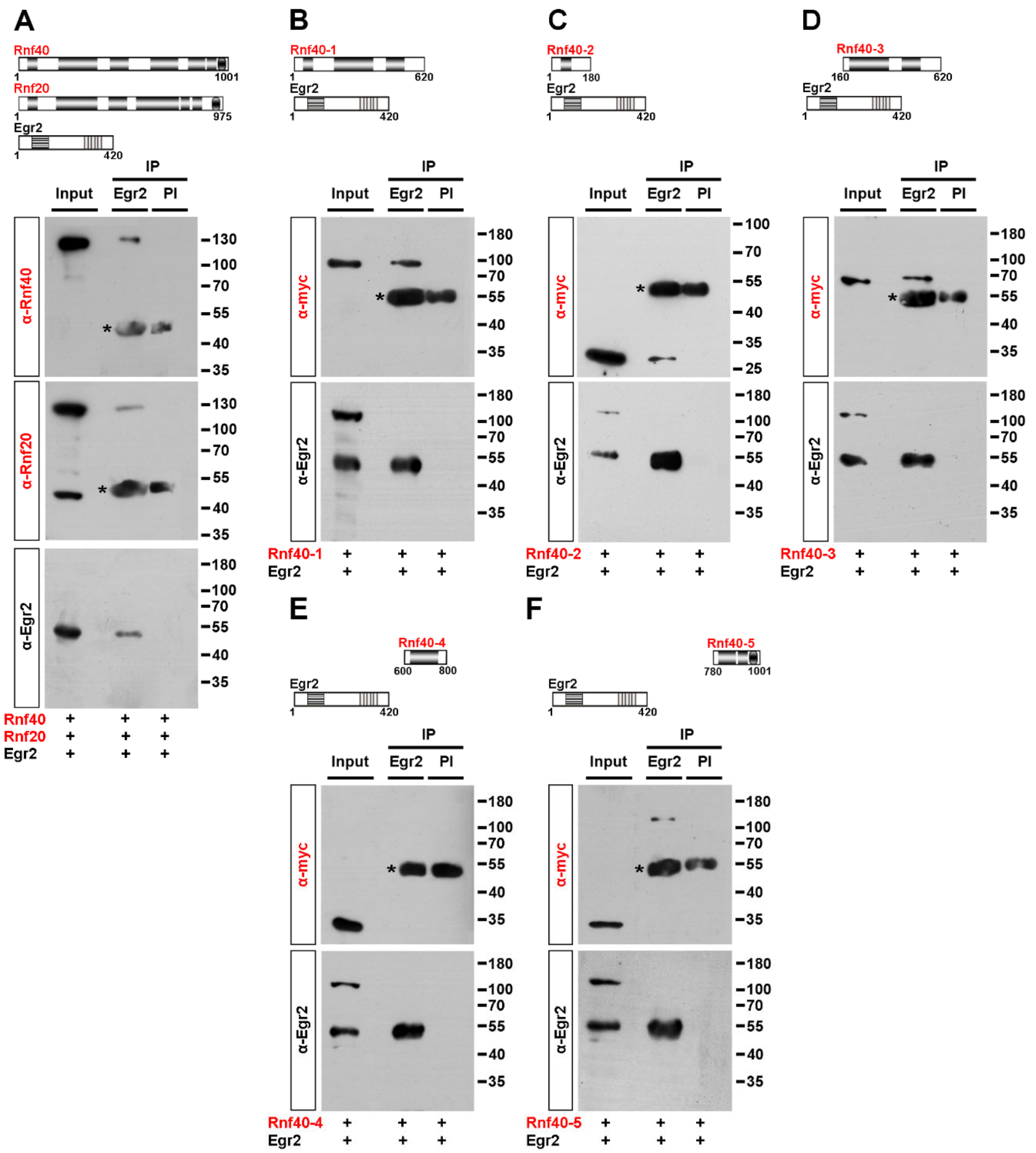

Supplementary Figure S9: Physical interaction between Egr2, Rnf40 and Rnf20 (part 1). (**A-F**) Uncropped western blots documenting co-immunoprecipitation (IP) of full length Rnf40 and full length Rnf20 (A) or myc-tagged Rnf40 truncated versions as depicted above the panels (B-F) with antibodies directed against Egr2 from extracts of HEK293T cells transfected with expression plasmids as indicated below the panels. Precipitation with preimmune serum (PI) served as control. Asterisks marks unspecific bands of Egr2 antibody heavy chains. Numbers on the right of panels indicate position of co-electrophoresed size markers in kDa.

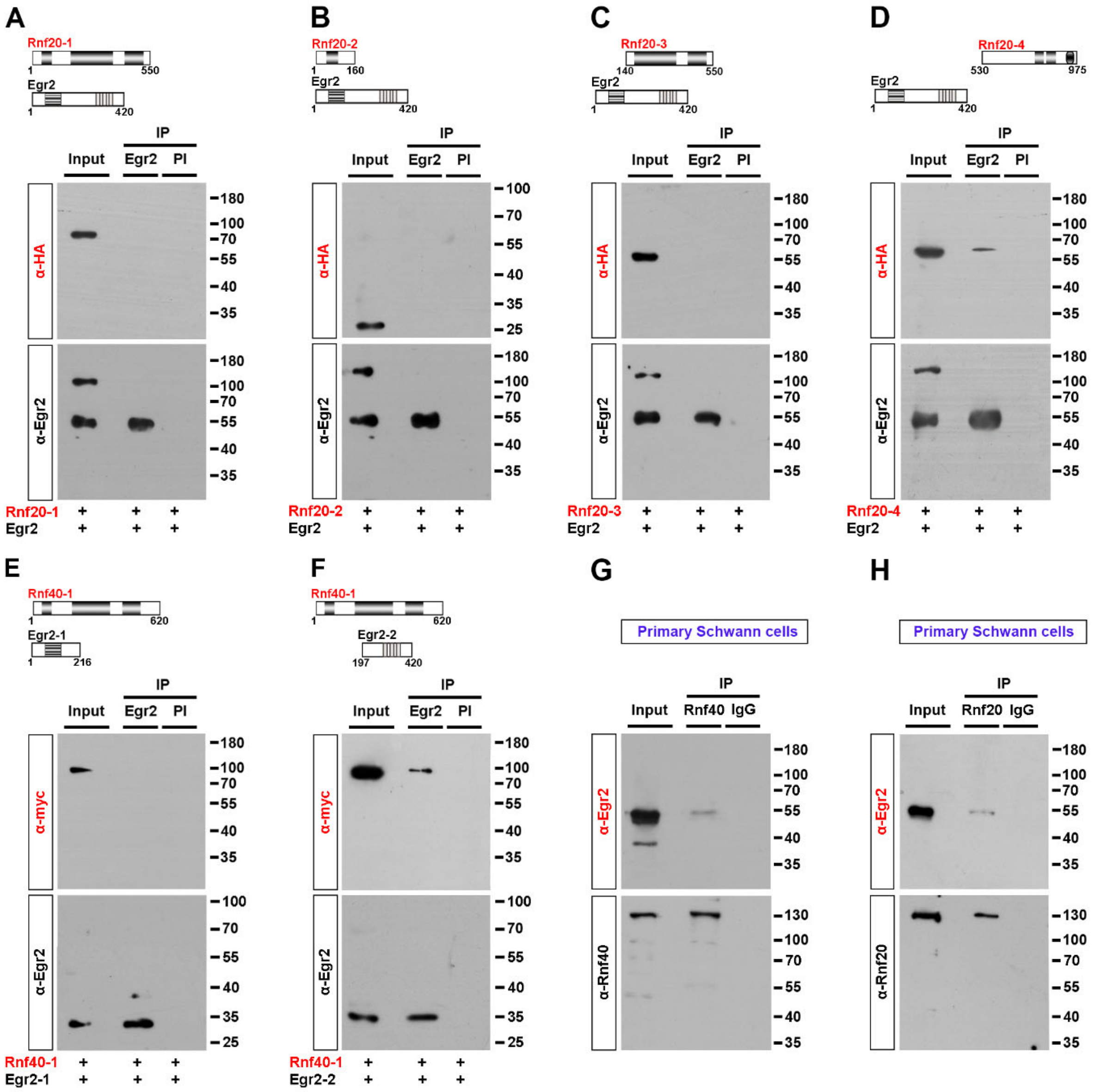

Supplementary Figure S10: Physical interaction of Egr2, Rnf40 and Rnf20 (part 2). (**A-D**) Uncropped western blots documenting co-immunoprecipitation (IP) of HA-tagged Rnf20 truncated versions as depicted above the panels with antibodies directed against Egr2 from extracts of HEK293T cells transfected with expression plasmids as indicated below the panels. (**E,F**) Uncropped western blots documenting co-immunoprecipitation (IP) of Egr2 truncated versions as depicted above the panels with antibodies directed against myc-tagged Rnf40 from extracts of HEK293T cells transfected with expression plasmids as indicated below the panels. Precipitation with preimmune serum (PI) served as control. (**G,H**) Uncropped western blots documenting co-immunoprecipitation of Egr2 with antibodies directed against Rnf40 (G) and Rnf20 (H) from extracts of differentiating SC cultures. Precipitation with IgG served as control. Numbers on the right of panels indicate position of co-electrophoresed size markers in kDa.

SUPPLEMENTARY TABLES

Percentage of Rnf40-positive SCs in control and Rnf40$^{\Delta SC}$ mice (Fig. 1M)

Age	Genotype	Mean value ± SEM
P0	control	100.00 ± 0
	Rnf40ΔSC	0.00 ± 0

Number of Dapi-positive cells in sciatic nerves of control and Rnf40$^{\Delta SC}$ mice (Fig. 2, Supplementary Fig. S8)

Marker	Age	Genotype	Mean value ± SEM
Dapi	P0	control	256.22 ± 8.75
		Rnf40ΔSC	240.32 ± 7.23
	P5	control	527.15 ± 17.21
		Rnf40ΔSC	492.76 ± 23.43
	P14	control	555.00 ± 19.41
		Rnf40ΔSC	632.20 ± 27.62
	P28	control	474.75 ± 17.13
		control	479.10 ± 6.72
		Rnf40ΔSC	548.88 ± 23.58
		Rnf40ΔSC	554.10 ± 22.75
		dko	563.90 ± 18.67
	P56	control	420.25 ± 27.38
		Rnf40ΔSC	518.92 ± 11.62

Percentage of marker-positive cells in sciatic nerves of control and Rnf40$^{\Delta SC}$ mice (Fig. 2, Fig. 3, Supplementary Fig. S8)

Marker	Age	Genotype	Mean value ± SEM
Sox10	P0	control	74.22 ± 1.23
		Rnf40ΔSC	73.82 ± 1.12
	P5	control	67.60 ± 1.12
		Rnf40ΔSC	69.55 ± 0.64
	P14	control	67.08 ± 1.11
		Rnf40ΔSC	68.64 ± 1.43
	P28	control	54.33 ± 1.05
		Rnf40ΔSC	55.52 ± 3.22
		dko	58.98 ± 0.67
	P56	control	57.46 ± 1.27
		Rnf40ΔSC	56.45 ± 2.00
Ki67	P0	control	6.99 ± 0.95
		Rnf40ΔSC	11.50 ± 0.81
	P5	control	3.10 ± 0.59
		Rnf40ΔSC	10.43 ± 1.90
	P14	control	2.15 ± 0.51
		Rnf40ΔSC	4.88 ± 0.57

	P28	control	1.01 ± 0.16
		Rnf40ΔSC	4.00 ± 0.12
	P56	control	0.48 ± 0.17
		Rnf40ΔSC	3.20 ± 0.12
Ki67 + Sox10	P0	control	8.19 ± 0.48
		Rnf40ΔSC	15.38 ± 1.21
	P5	control	1.72 ± 0.42
		Rnf40ΔSC	6.61 ± 0.58
	P14	control	1.58 ± 0.43
		Rnf40ΔSC	4.32 ± 0.24
	P28	control	0.37 ± 0.19
		Rnf40ΔSC	2.97 ± 0.06
	P56	control	0.23 ± 0.13
		Rnf40ΔSC	1.95 ± 0.09
TUNEL	P5	control	0.10 ± 0.06
		Rnf40ΔSC	0.14 ± 0.04
	P14	control	0.28 ± 0.10
		Rnf40ΔSC	0.10 ± 0.01
	P28	control	0.16 ± 0.11
		Rnf40ΔSC	0.18 ± 0.09
	P56	control	0.00 ± 0.00
		Rnf40ΔSC	0.15 ± 0.08
Sox2	P0	control	20.34 ± 0.47
		Rnf40ΔSC	27.78 ± 2.62
	P5	control	13.72 ± 0.40
		Rnf40ΔSC	31.37 ± 1.04
	P14	control	7.95 ± 0.59
		Rnf40ΔSC	29.23 ± 0.91
	P28	control	2.77 ± 0.82
		Rnf40ΔSC	25.18 ± 1.04
		dko	0.0 ± 0.0
	P56	control	1.30 ± 0.93
		Rnf40ΔSC	20.44 ± 1.19
Oct6	P0	control	50.46 ± 0.84
		Rnf40ΔSC	50.78 ± 0.91
	P5	control	56.85 ± 5.29
		Rnf40ΔSC	60.75 ± 3.65
	P14	control	35.42 ± 2.40
		Rnf40ΔSC	56.18 ± 1.65
	P28	control	15.44 ± 1.54
		Rnf40ΔSC	51.71 ± 1.48
		dko	50.18 ± 0.94
	P56	control	15.54 ± 3.91
		Rnf40ΔSC	45.64 ± 2.48
Egr2	P0	control	23.22 ± 0.85
		Rnf40ΔSC	27.26 ± 2.25

	P5	control	43.10 ± 0.86
		Rnf40ΔSC	45.34 ± 1.03
	P14	control	47.25 ± 1.73
		Rnf40ΔSC	49.87 ± 1.22
	P28	control	50.00 ± 1.84
		Rnf40ΔSC	43.86 ± 2.56
	P56	control	48.58 ± 2.29
		Rnf40ΔSC	48.11 ± 1.82
Iba1	P0	control	1.80 ± 0.16
		Rnf40ΔSC	2.75 ± 0.34
	P5	control	2.67 ± 0.09
		Rnf40ΔSC	3.89 ± 0.13
	P14	control	2.63 ± 0.55
		Rnf40ΔSC	5.15 ± 1.27
	P28	control	2.19 ± 0.58
		Rnf40ΔSC	9.12 ± 1.22
		dko	7.23 ± 1.23
	P56	control	2.81 ± 0.21
		Rnf40ΔSC	8.87 ± 0.67

Percentage of unmyelinated large caliber axons in sciatic nerves of control and Rnf40$^{\Delta SC}$ mice (Fig. 4R)

Age	Genotype	Mean value ± SEM
P14	control	0.00 ± 0.00
	Rnf40ΔSC	37.05 ± 2.44
P28	control	0.00 ± 0.00
	Rnf40ΔSC	41.05 ± 4.00
P56	control	0.00 ± 0.00
	Rnf40ΔSC	80.08 ± 4.52
P168	control	0.00 ± 0.00
	Rnf40ΔSC	78.26 ± 1.34

Numbers of large caliber axons in tibial branch of sciatic nerves of control and Rnf40$^{\Delta SC}$ mice (Fig 4T)

Age	Genotype	Mean value ± SEM
P14	control	2945.38 ± 25.28
	Rnf40ΔSC	2941.99 ± 103.22
P28	control	2954.74 ± 75.72
	Rnf40ΔSC	2227.63 ± 38.01
P56	control	2879.66 ± 27.08
	Rnf40ΔSC	2031.43 ± 15.62
P168	control	2961.02 ± 52.37
	Rnf40ΔSC	1784.58 ± 53.36

G-ratio of myelin sheaths in sciatic nerves of control and Rnf40$^{\Delta SC}$ mice
(Fig. 4S, Supplementary Fig. 4I-L)

Age	Genotype	Mean value ± SEM
P14	control	0.58 ± 0.01
	<1.5µm Ø axons	0.61 ± 0.01
	1.5-2.0µm Ø axons	0.57 ± 0.01
	2.0-2.5µm Ø axons	0.56 ± 0.01
	2.5-3.0µm Ø axons	0.57 ± 0.02
	Rnf40ΔSCs	0.73 ± 0.01
	<1.5µm Ø axons	0.70 ± 0.02
	1.5-2.0µm Ø axons	0.74 ± 0.01
	2.0-2.5µm Ø axons	0.75 ± 0.01
	2.5-3.0µm Ø axons	0.74 ± 0.01
P28	control	0.53 ± 0.01
	<1.5µm Ø axons	0.55 ± 0.01
	1.5-2.0µm Ø axons	0.52 ± 0.01
	2.0-2.5µm Ø axons	0.51 ± 0.01
	2.5-3.0µm Ø axons	0.52 ± 0.02
	>3.0µm Ø axons	0.54 ± 0.01
	Rnf40ΔSCs	0.76 ± 0.01
	<1.5µm Ø axons	0.73 ± 0.01
	1.5-2.0µm Ø axons	0.75 ± 0.01
	2.0-2.5µm Ø axons	0.78 ± 0.01
	2.5-3.0µm Ø axons	0.80 ± 0.02
	>3.0µm Ø axons	0.85 ± 0.01
P56	control	0.58 ± 0.01
	<1.5µm Ø axons	0.58 ± 0.01
	1.5-2.0µm Ø axons	0.57 ± 0.01
	2.0-2.5µm Ø axons	0.58 ± 0.01
	2.5-3.0µm Ø axons	0.60 ± 0.01
	>3.0µm Ø axons	0.60 ± 0.01
	Rnf40ΔSCs	0.83 ± 0.01
	<1.5µm Ø axons	0.75 ± 0.04
	1.5-2.0µm Ø axons	0.86 ± 0.01
	2.0-2.5µm Ø axons	0.83 ± 0.01
	2.5-3.0µm Ø axons	0.86 ± 0.01
	>3.0µm Ø axons	0.90 ± 0.01
P168	control	0.59 ± 0.01
	<2µm Ø axons	0.58 ± 0.01
	2.0-2.5µm Ø axons	0.57 ± 0.02
	2.5-3.0µm Ø axons	0.59 ± 0.01
	3.0-3.5µm Ø axons	0.60 ± 0.01
	>3.5µm Ø axons	0.61 ± 0.01
	Rnf40ΔSCs	0.84 ± 0.03
	<2µm Ø axons	0.75 ± 0.06

	2.0-2.5µm Ø axons	0.84 ± 0.01
	2.5-3.0µm Ø axons	0.85 ± 0.01
	3.0-3.5µm Ø axons	0.88 ± 0.01
	>3.5µm Ø axons	0.91 ± 0.01

Genes with changed expression in Rnf40$^{\Delta SC}$ sciatic nerves (Fig. 5D,E,G,H)

Cellular Process	Gene	log2 fold change ± SEM	adjusted P-value
myelination	Gjb1	-1,78 ± 0,18	< 0.0001
	Mag	-1.95 ± 0.12	< 0.0001
	Mal	-1.61 ± 0.08	< 0.0001
	Mbp	-1.61 ± 0.07	< 0.0001
	Mpz	-2.12 ± 0.07	< 0.0001
	Nfasc	-0.77 ± 0.08	< 0.0001
	Pmp22	-2.11 ± 0.07	< 0.0001
	Prx	-1.58 ± 0.06	< 0.0001
lipid biosynthesis	Acss1	-2.62 ± 0.23	< 0.0001
	Fa2h	-2.30 ± 0.10	< 0.0002
	Fasn	-2.05 ± 0.09	< 0.0003
	Hmgcr	-2.08 ± 0.10	< 0.0004
	Hsd17b7	-2.30 ± 0.22	< 0.0005
	Lss	-3.43 ± 0.28	< 0.0006
	Msmo1	-2.59 ± 0.12	< 0.0007
cell adhesion	Cadm1	1.28 ± 0.12	< 0.0001
	Cdh10	1.12 ± 0.30	0,0001
	Chl1	0.98 ± 0.15	< 0.0001
	Col7a1	1.21 ± 0.22	< 0.0001
	Jam3	0.97 ± 0.11	< 0.0001
	Lama1	3.96 ± 0.37	< 0.0001
	Ncan	4.14 ± 0.37	< 0.0001
stage-specific TFs	Pax3	0.00 ± 0.00	N/A
	Tfap2a	-0.19 ± 0.40	0,8365
	cJun	0.11 ± 0.15	0,7202
	Hes5	-0.12 ± 0.21	0,5542
	Hey1	-0.11 ± 0.27	0,8672
	Hey2	-0.49 ± 0.45	0,5578
	Zeb2	-0.32 ± 0.08	0,0016
	Sox2	1.77 ± 0.28	< 0.0001
	Brn2	1.76 ± 0.17	< 0.0001
	Oct6	2.02 ± 0.12	< 0.0001

Expression plasmids for Rnf40, Rnf20 and Egr2 (Fig.7, Supplementary Fig. S9 & S10)

Plasmid/Fragment	Amino acids	Tag	Contained domains	Mol. weight (kDa)
pCMV5-Rnf40	1-1001	myc	coiled coil, RING finger	130
pCMV5-Rnf40-1	1-620	myc	coiled coil	90
pCMV5-Rnf40-2	1-180	myc	coiled coil	25
pCMV5-Rnf40-3	160-620	myc	coiled coil	65
pCMV5-Rnf40-4	600-800	myc	coiled coil	30
pCMV5-Rnf40-5	780-1001	myc	coiled coil, RING finger	32
pCMV5-Rnf20	1-975	HA	coiled coil, RING finger	125
pCMV5-Rnf20-1	1-550	HA	coiled coil	70
pCMV5-Rnf20-2	1-160	HA	coiled coil	25
pCMV5-Rnf20-3	140-550	HA	coiled coil	55
pCMV5-Rnf20-4	530-975	HA	coiled coil, RING finger	65
pCMV5-Egr2	1-420	/	DUF3446, C2H2 Zinc finger	50
pCMV5-Egr2-1	1-216	/	DUF3446	27
pCMV5-Egr2-2	197-420	/	C2H2 Zinc finger	30

Determination of endogenous transcript levels of selected myelin genes in Neuro2a cells transfected with various combinations of expression plasmids (Fig. 7J-M)

Gene	Primer	Effector	Mean ± SEM
Mbp	5'-CCAAGTTCACCCCTACTCCA-3'	-	1.00 ± 0.02
	5'-TAAGTCCCCGTTTCCTGTTG-3'	Egr2	2.90 ± 0.33
		shScr	1.06 ± 0.08
		shRnf40	1.02 ± 0.13
		Egr2 + shScr	3.23 ± 0.03
		Egr2 + shRnf40	1.23 ± 0.11
Mpz	5’-CTGGTCCAGTGAATGGGTCT-3’	-	1.00 ± 0.03
	5’-CATGTGAAAGTGCCGTTGTC-3’	Egr2	3.33 ± 0.50
		shScr	0.97 ± 0.12
		shRnf40	0.98 ± 0.02
		Egr2 + shScr	3.53 ± 0.20
		Egr2 + shRnf40	1.75 ± 0.12
Mag	5'-TCACCAGCATCCTCACGATC-3'	-	1.00 ± 0.06
	5'-AATCAGGATGGCAAAGGCGA-3'	Egr2	34.63 ± 1.67
		shScr	1.08 ± 0.24
		shRnf40	1.13 ± 0.11
		Egr2 + shScr	33.61 ± 5.20
		Egr2 + shRnf40	13.37 ± 3.14
Prx	5’-TAGTGGGTGAGGGCATCTTC-3’	-	1.00 ± 0.01
	5’-TGGTGACGTGAGTTCCACAT-3’	Egr2	4.12 ± 0.37
		shScr	1.02 ± 0.15
		shRnf40	0.94 ± 0.02
		Egr2 + shScr	4.23 ± 0.14
		Egr2 + shRnf40	1.79 ± 0.06
